# Supplementary figures and images for: Chemogenetic attenuation of neuronal activity in the entorhinal cortex reduces Aβ and tau pathology in the hippocampus
Source: PLoS Biol. 2020 Aug 21;18(8):e3000851. doi: 10.1371/journal.pbio.3000851 (PMC7467290; doi:10.1371/journal.pbio.3000851)

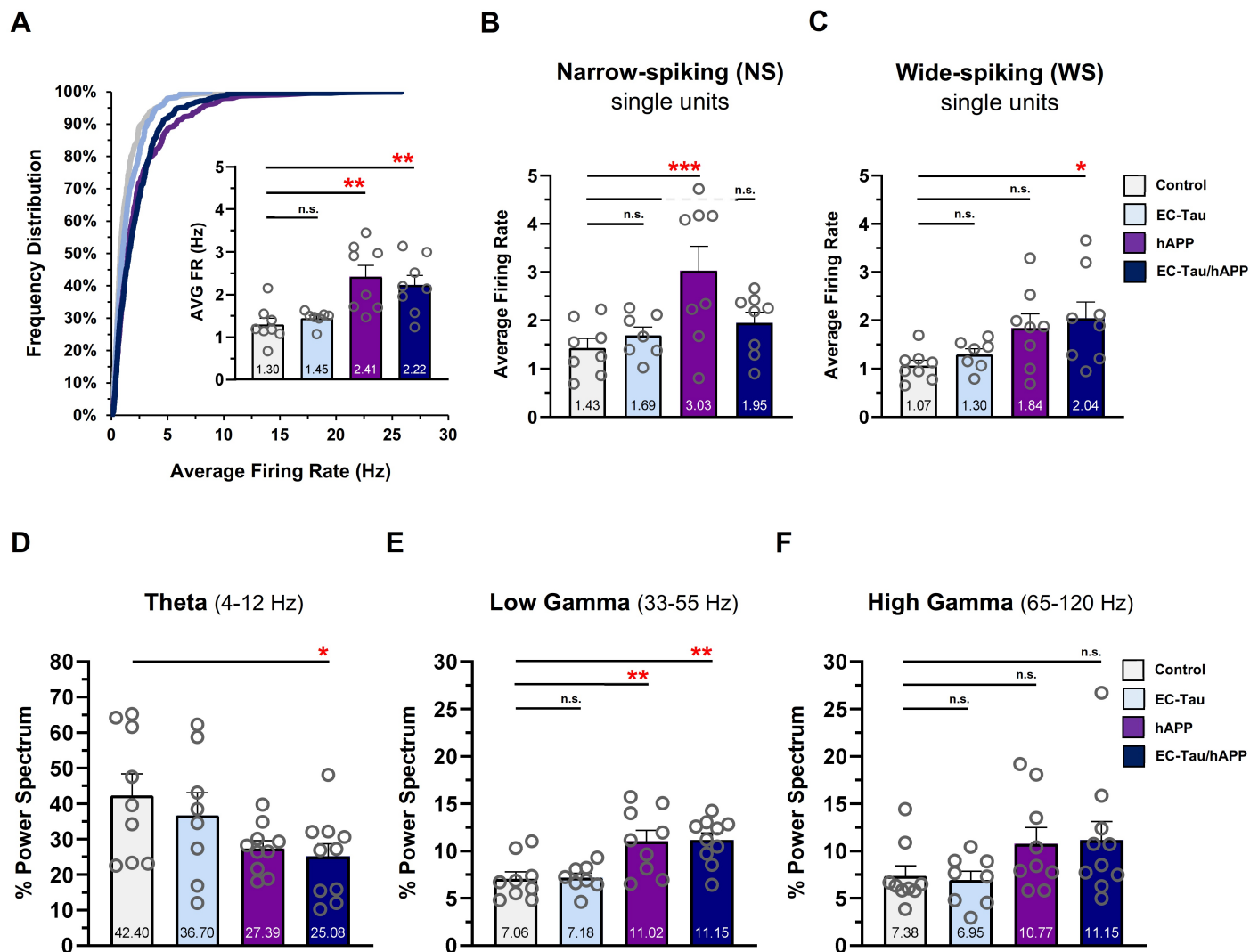

Supplement: S1 Fig — A minimum–maximum speed filter was applied to the electrophysiology datasets post hoc to remove single-unit spikes and LFP activity that occurred during bouts of behavioral immobility. For details, please see Materials and methods. A. The cumulative frequency distributions of speed-filtered EC neuronal firing rates for each genotype are shown. Distributions from EC-Tau/hAPP (n = 588 cells) and hAPP (n = 532 cells) mice were shifted toward increased firing rates compared with control (n = 386 cells). Two-sample Kolmogorov–Smirnov test: EC-Tau/hAPP versus control: D = 0.268, p < 0.001; hAPP versus control: D = 0.248, p < 0.001. Insert, speed-filtered firing rates were significantly increased in EC-Tau/hAPP mice and hAPP mice versus Control. One-way ANOVA: p < 0.001. Dunnett’s multiple comparisons tests, p < 0.01. B-C. NS and WS cells were examined by genotype after speed filtering. hAPP mice exhibited increased NS neuronal firing rates versus Control. One-way ANOVA: p < 0.01. Dunnett’s multiple comparisons tests, p < 0.01. EC-Tau/hAPP mice exhibited increased WS neuronal firing rates versus control. One-way ANOVA: p < 0.05. Dunnett’s multiple comparisons tests, p < 0.05. D. Speed-filtered percentage theta power values were significantly reduced in EC-Tau/hAPP mice compared to Control. One-way ANOVA: p < 0.05. Dunnett’s multiple comparisons tests, p < 0.05. E. Speed-filtered percentage low-gamma power values were significantly increased in EC-Tau/hAPP and hAPP mice versus control. One-way ANOVA: p < 0.001. Dunnett’s multiple comparisons tests, p < 0.01. F. No differences were detected across genotype in speed-filtered percentage high gamma power. Bar graphs represent mean ± SEM. Individual values per mouse appear as overlays. *p < 0.05; **p < 0.01; ***p < 0.001. Source data are available in S1 Data. EC, entorhinal cortex; hAPP, human amyloid precursor protein; LFP, local field potential; NS, narrow-spiking; SEM, standard error mean; WS, wide-spiking. (PDF) [file pbio.3000851.s002.pdf]

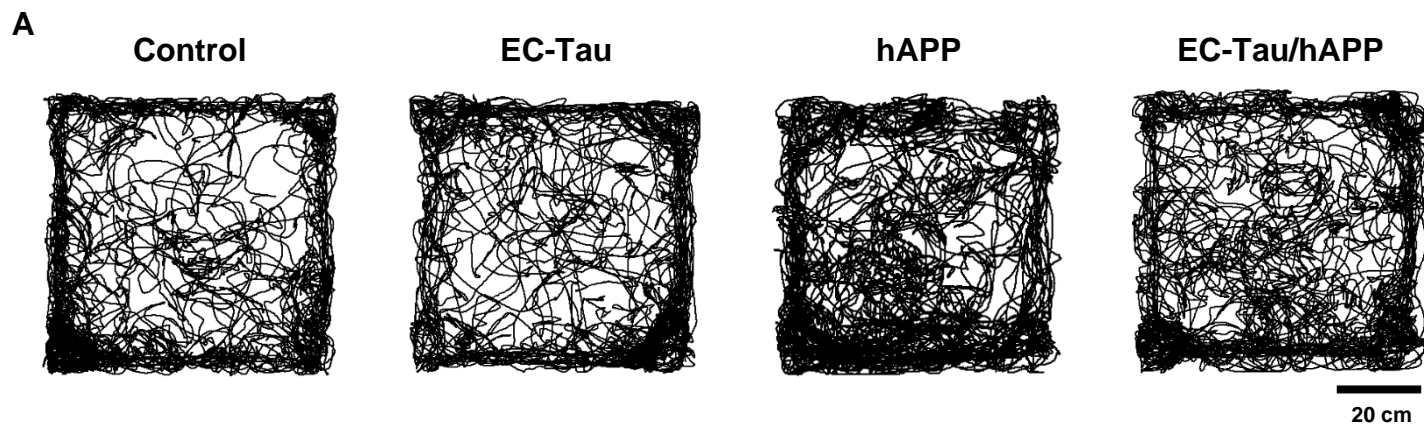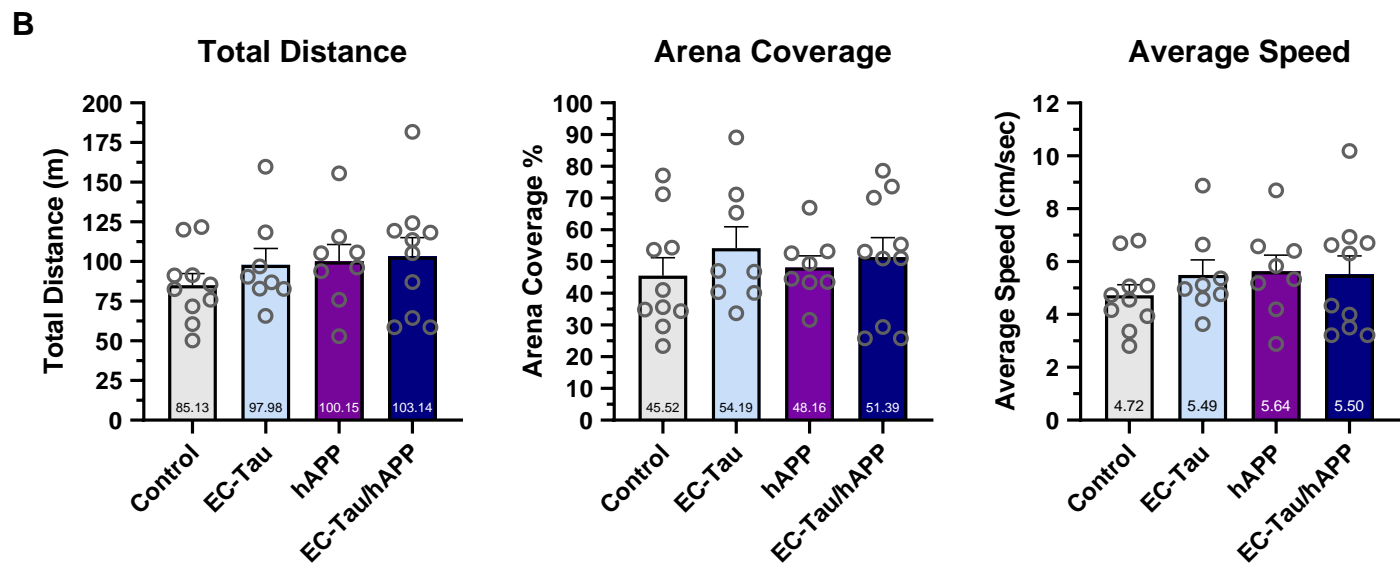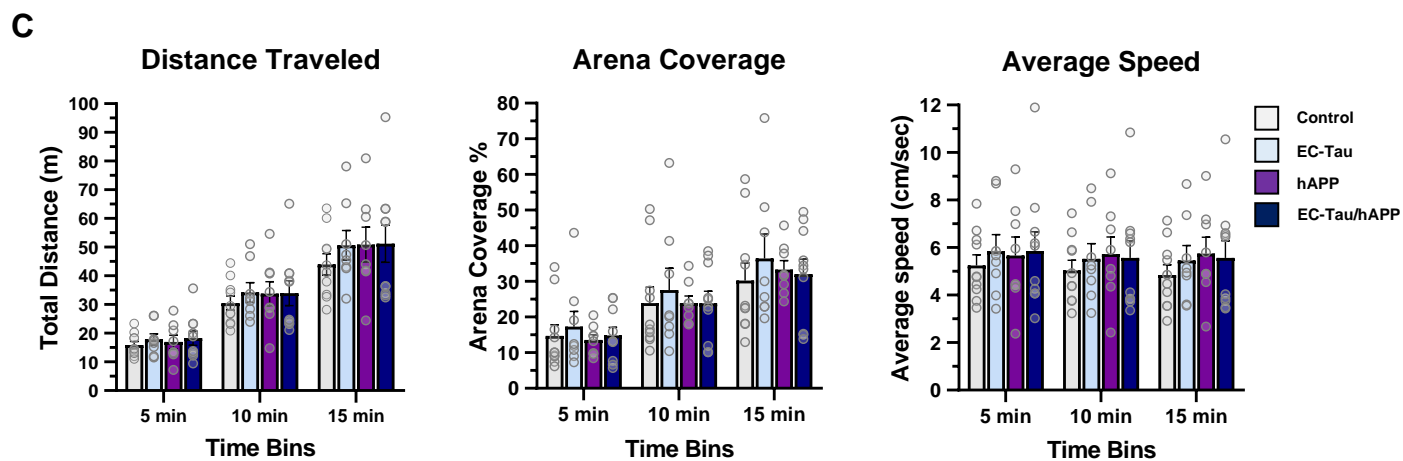

Supplement: S2 Fig — Locomotor activity was assessed in vivo by analyzing the position data of each mouse during active exploration in an open field. Sample sizes are as follows: Control, n = 10 total: n = 6 female, n = 4 male; EC-Tau, n = 8 total: n = 4 female, n = 4 male; hAPP, n = 8 total: n = 4 female, n = 4 male; EC-Tau/hAPP, n = 10 total: n = 6 female, n = 4 male. A. Representative trajectories are shown for one recording session per genotype. Scale bar, 20cm. B. The following parameters in the open field were analyzed and compared across genotype: the total distance traveled (m), the % arena coverage and average speed (cm/second). No significant group differences were detected on any measure. One-way ANOVA tests: Total distance (meter), F(3,32) = 0.228, p > 0.05; % of arena coverage, F(3,32) = 0.345, p > 0.05; average speed (cm/second), F(3,32) = 0.237, p > 0.05. C. No significant group differences in behavioral measures were detected in the first 5-, 10-, or 15-minute time-bins of the recording sessions. Linear mixed-effects analyses were performed for each dependent measure. Bar graphs represent mean ± SEM. Individual mouse values appear as overlays and represent the mean of 3 averaged recording sessions per mouse. Source data are available in S1 Data. Representative Axona.pos files for each mouse are available at: https://github.com/HussainiLab/PLOS-Biology-manuscript-data. Aβ, amyloid-beta; EC, entorhinal cortex; hAPP, human amyloid precursor protein; SEM, standard error mean. (PDF) [file pbio.3000851.s003.pdf]

**A****Acute hM4Di EC DREADDs activation**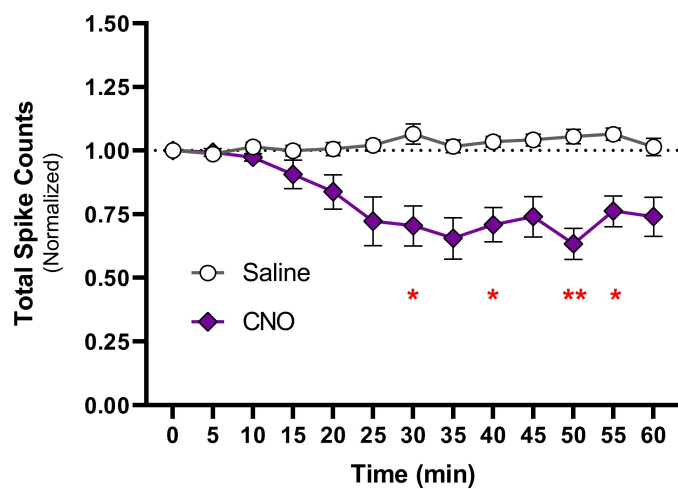**B****Acute hM4Di EC DREADDs activation**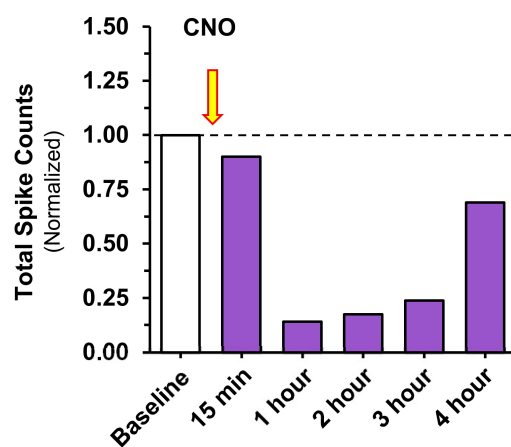**C**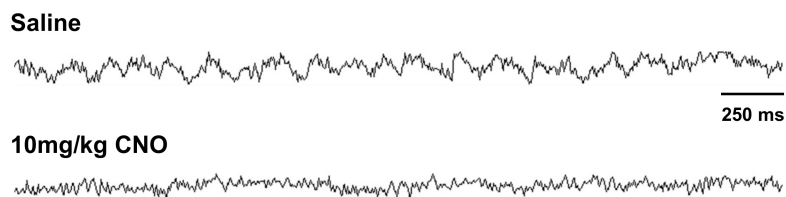**D****Theta (4-12 Hz)**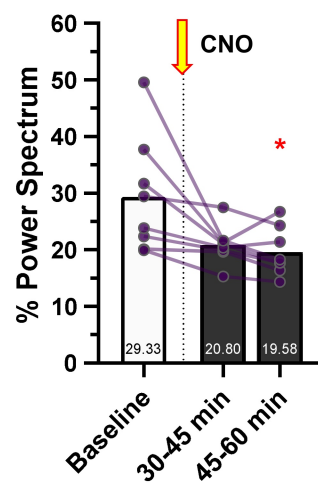

Supplement: S3 Fig — Single injections of CNO (i.p.) were used to determine salient recording measures of EC DREADDs activation in vivo. Long-term recordings were performed in 12- to 16-month mice to first determine the onset and duration of altered single-unit activity after hM4Di EC DREADDs activation. A. Averaged total spike counts are shown for 60 min recordings following 5–10 mg/kg CNO and Saline injections (n = 6 mice: n = 1 female, n = 5 male). Spike counts were first normalized to individual mouse baseline measures collected prior to drug treatment and then plotted in 5-minute time-bins for 60 minutes total. hM4Di EC DREADDs activation begins to affect total spike counts approximately 20 minutes after injection, with significant post hoc comparisons evident at 40 minutes (p < 0.05), 50 minutes (p < 0.01), and 55 minutes (p < 0.05) time-bins versus Saline. Repeated-measures 2-way ANOVA with Greenhouse–Geisser correction: (time × drug treatment) F(11,110) = 5.448, p < 0.001. (Time) F(2.4,23.5) = 3.112, p >0.05. (Drug Treatment) F(1,10) = 30.490, p <0.001. Sidak’s multiple comparisons test. Diamond (purple), hM4Di EC DREADDs; circle (white), aaline. B. Total spike counts (normalized to baseline) following hM4Di EC DREADDs activation are shown for one 4-hour recording session in a 16-month hAPP mouse. Mean spiking activity in 15 min time-bins in shown. For 1- to 4-hour time points, the last 15 minutes of each hour was analyzed. C. Acute hM4Di EC DREADDs activation measurably impacted percentage theta power in vivo. Representative LFP traces are shown for both CNO and saline conditions. Notable differences in EC theta modulation are shown after 10 mg/kg CNO treatment. D. hM4Di EC DREADDs activation decreased % theta power values compared to baseline, with a significant reduction detected at 45–60 minutes post-CNO (p < 0.05) (n = 8 mice: n = 4 female, n = 4 male). Repeated-measures ANOVA: F(2,7) = 7.079, p < 0.05. Dunnett’s multiple comparisons test: 45–60 minutes versus baseline, p < [file pbio.3000851.s004.pdf]
