# Supplementary material for: Chemogenetic attenuation of neuronal activity in the entorhinal cortex reduces Aβ and tau pathology in the hippocampus
Source: PLoS Biol. 2020 Aug 21;18(8):e3000851. doi: 10.1371/journal.pbio.3000851 (PMC7467290; doi:10.1371/journal.pbio.3000851)
Supplement: S4 Fig — Area measurements of hippocampal ROI downstream from hM4Di DREADDs-expressing EC (right hemisphere) were compared to contralateral hippocampal ROIs (left hemisphere). A. Top, 8-bit gray scale images of 6E10+ immunoreactivity in a horizontal brain section from a 16-month EC-Tau/hAPP mouse after chronic hM4Di EC DREADDs activation. A minimum threshold value was first applied to each image and then the ROIs were defined for right and left hippocampus (magenta). The % area of 6E10+ immunoreactivity above threshold was used to quantify hAPP/Aβ accumulation within the right and left hippocampal ROIs. Scale bars, 500μm. Bottom, Higher magnification of 6E10+ immunoreactivity within ROIs. Black pixels depict 6E10+ immunoreactivity above threshold on a white background. Scale bars, 250μm. B. Left, no differences were detected in right-versus-left hippocampal ROIs (mm2) in EC-Tau/hAPP mice after chronic hM4Di EC DREADDs activation. Paired t-test: t (8) = 1.422, p > 0.05. Right, no differences were detected in right-versus-left hippocampal ROIs in mice subjected to control conditions. Paired t-test: t (8) = 1.856, p > 0.05. Individual values represent the average of 3 sections per mouse and appear as colored bar overlays. Mean ROI area is depicted within bar graphs. C. A scatter plot of right-versus-left hippocampal ROIs (mm2) is shown for 6E10+ immunostained brain sections (total, n = 18 data coordinates; n = 3 sections/mouse; n = 18 mice). The coefficient of determination (R2 = 0.7593) is shown below the scatter. D. High-magnification images were taken at 20× for semiquantitative analysis of tau immunoreactivity within hippocampal subregions. MC1+ staining is shown for 1 EC-Tau/hAPP mouse after chronic hM4Di EC DREADDs activation. Top panel, 8-bit grayscale images of MC1+ immunoreactivity are shown for 3 hippocampal regions analyzed: DG, CA1, and the Sub. MC1+ immunoreactivity within the defined ROI (black outline) appears as red pixels above minimum threshold. Bottom panel, [file pbio.3000851.s005.pdf]

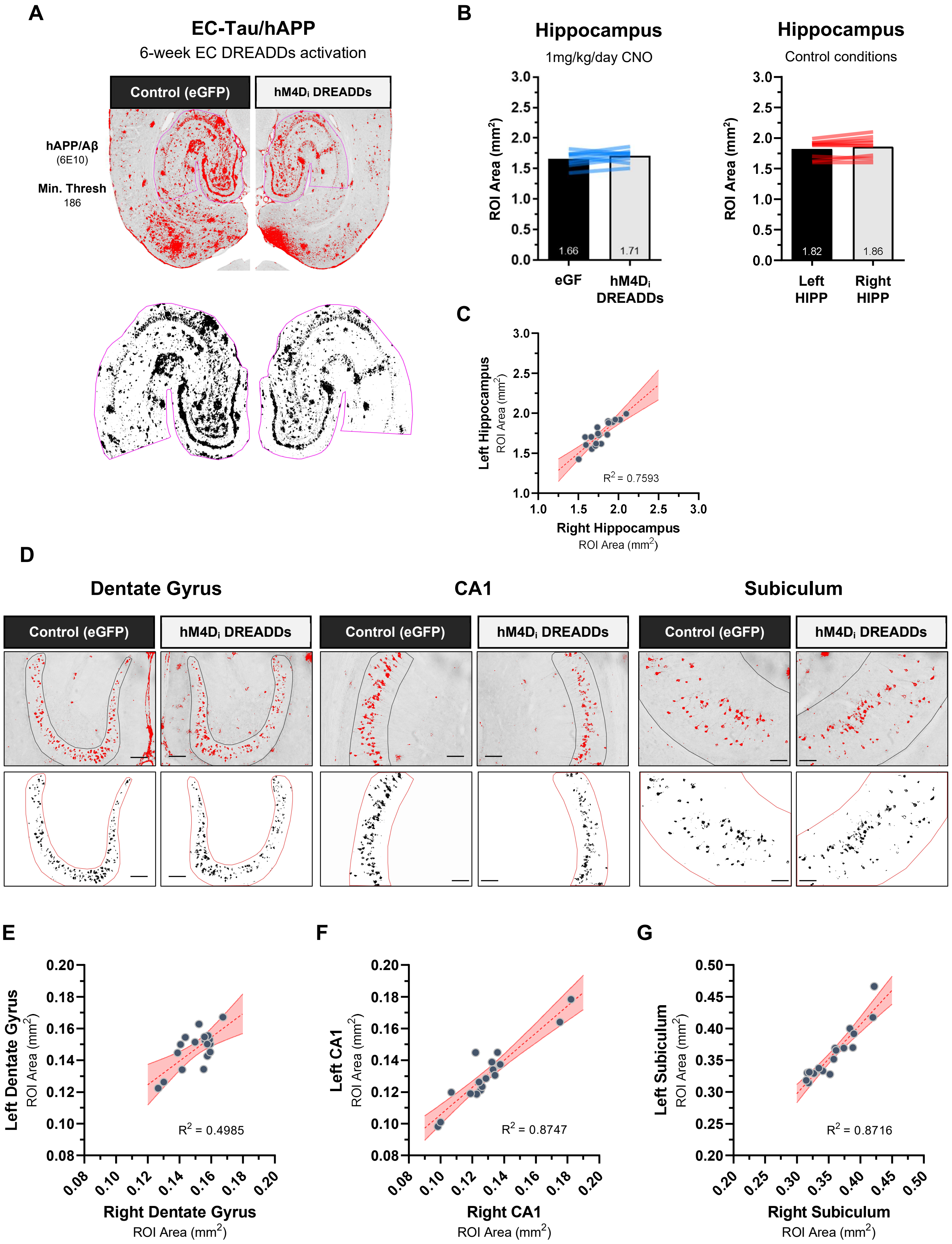
A
EC-Tau/hAPP
6-week EC DREADDs activation
Control (eGFP)
hM4Dᵢ DREADDs
hAPP/Aβ
(6E10)
Min. Thresh
186
B
Hippocampus
1mg/kg/day CNO
ROI Area (mm²)
1.66
1.71
eGF
hM4Dᵢ DREADDs
Hippocampus
Control conditions
1.82
1.86
Left HIPP
Right HIPP
C
Left Hippocampus
ROI Area (mm²)
R² = 0.7593
Right Hippocampus
ROI Area (mm²)
D
Dentate Gyrus
CA1
Subiculum
Control (eGFP)
hM4Dᵢ DREADDs
E
Left Dentate Gyrus
ROI Area (mm²)
R² = 0.4985
Right Dentate Gyrus
ROI Area (mm²)
F
Left CA1
ROI Area (mm²)
R² = 0.8747
Right CA1
ROI Area (mm²)
G
Left Subiculum
ROI Area (mm²)
R² = 0.8716
Right Subiculum
ROI Area (mm²)
