# Supplementary material for: Chemogenetic attenuation of neuronal activity in the entorhinal cortex reduces Aβ and tau pathology in the hippocampus
Source: PLoS Biol. 2020 Aug 21;18(8):e3000851. doi: 10.1371/journal.pbio.3000851 (PMC7467290; doi:10.1371/journal.pbio.3000851)
Supplement: Attachment — Submitted filename: Rodriguez et al - PLOS Biology - Response to Reviewers.pdf [file pbio.3000851.s006.pdf]

POINT BY POINT RESPONSES

Reviewer #1

In this manuscript, Rodriguez et al. report that tau pathology in the EC-HIPP network is enhanced in mouse models expressing both EC-Tau and hAPP compared to models only expressing EC-Tau. Second, they investigate neuronal activity in the EC of freely moving mice using extracellular electrophysiology and report increased neuronal activity in hAPPexpressing mice. Finally, the authors developed a methodology to chemogenetically decrease neuronal activity and investigated the effects of this manipulation on Aβ-and tau pathology in the EC-HIPP network. If true, the study by Rodriguez et al. raises provides some interesting insights in the pathology of Alzheimer's disease, but there are some major concerns that they need to address.

Major

1. The number of samples/experiments is often insufficient. In some cases (e.g. Fig. S2), the authors only use one mouse (n=1!), which is unacceptable.

We are happy to report that we have now included new data from additional mice to studies throughout this revised manuscript, resulting in increased sample sizes and more robust statistical analyses on a per mouse basis. For instance, please see revised Supplemental Table 1 for a breakdown of the single-unit electrophysiology data (n=31 mice total).

To specifically address Reviewer #1's concerns with former Figure S2, we updated the first panel to include data from n=6 mice that were given an acute injection of high dose, 5-10mg/kg CNO to activate hM4Di EC DREADDs (updated Supplemental Figure 3A). We show that acute hM4Di EC DREADDs activation (purple diamonds) results in decreased total spike counts in the EC compared to when the same mice were administered Saline injections (white circles). A two-way ANOVA with repeated measures was performed with Drug Treatment and Time bin as independent variables, followed by Sidak's multiple comparisons test for *post hoc* analyses. Panel B shows that spike counts remain lowered in one representative mouse for at least 4 hr. These data provided us a metric with which to track CNO-DREADDs mediated changes to neuronal activity *in vivo* (updated Figure 4A).

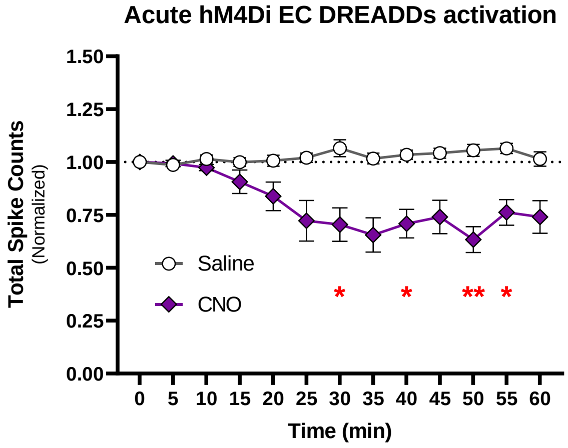

Two-way ANOVA w/ repeated measures

Interaction	$F_{(11,110)} = 5.448$	$p < 0.001$
Time	$F_{(2.4,23.5)} = 3.112$	$p > 0.05$
Drug	$F_{(1,10)} = 30.490$	$p < 0.001$

Sidak's multiple comparisons test

CNO vs Control, 30 min; $p < 0.05$
CNO vs Control, 40 min; $p < 0.05$
CNO vs Control, 50 min; $p < 0.01$
CNO vs Control, 45 min; $p < 0.05$

In addition, updated Supplemental Figure 3D shows that acute hM4Di EC DREADDs activation reliably reduces % Theta power within the LFP of n=8 mice, providing another useful metric in which to track chronic hM4Di EC DREADDs activation *in vivo* (updated Figure 4B). A repeated measures one-way ANOVA was performed on 15 min time bins (30-45 min, 45-60 min) and compared to Baseline (15 min prior to CNO injection) using Dunnett's multiple comparison test.

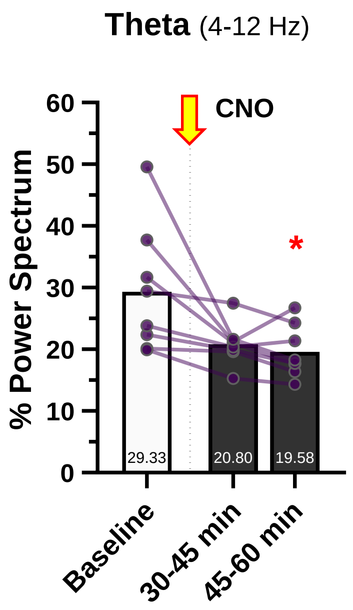

Repeated measures one-way ANOVA
$F_{(2,7)}$ = 7.079, $p < 0.05$

Dunnett's multiple comparisons test
45-60 min vs Baseline, $p < 0.05$

2. Furthermore, they assign mice of different genotypes (e.g. fig. 5) to the same statistical, which, again, is unacceptable, as the different genotypes may (and in fact do) have different properties.

We agree with the reviewer and have separated the genotypes where necessary. We have now reanalyzed our immunostaining data after the addition of new experimental mice that were added to our study. These data are now reported in updated Figures 4C-F and Figure 5, which show reduced hAPP/Aβ and tau marker staining in the hippocampus after chronic attenuation of EC neuronal activity. In Figure 4E, we discarded the data from hAPP/J20 (n=3) mice and added 6E10+ immunostaining data from five EC-Tau/hAPP mice, bringing the total sample size to n=9 with no mixed genotypes. We performed a paired *t* test on the left and right hemisphere data and found reduced 6E10+ immunoreactivity in the right hemisphere of these mice (Paired *t* test: $t(8)=5.919$, $p < 0.001$). To demonstrate that this reduction was due to DREADDs activation, we performed the same analysis on tissue sections from n=9 mice in our control conditions (Figure 4F). We did not detect hemispheric differences in 6E10+ immunoreactivity in the hippocampus of these mice (Paired *t* test: $t(8)=1.357$, $p = 0.212$).

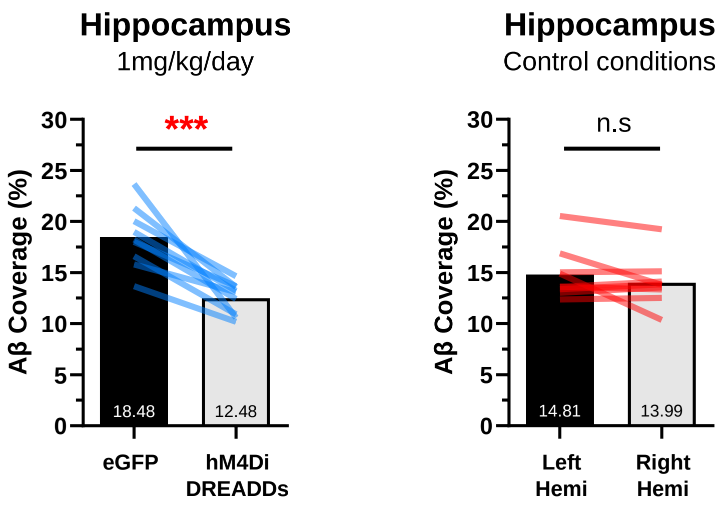

In Figure 5, we show higher resolution images of low magnification and higher magnification images of tau marker immunoreactivity in horizontal brain sections. We have now removed EC-Tau mice from our sample and performed semi-quantitative analyses on hM4Di EC DREADDs expressing EC-Tau/hAPP mice (n=6) administered

chronic administration of CNO (1 mg/kg/day). Data values are now shown for each mouse and for each marker in three hippocampal subregions (DG, CA1 and Subiculum), eliminating pseudo-replication from this figure. Boxplots from Figure 5J are shown to illustrate the updated analysis in our manuscript. One sample *t* tests: DG, $t_{(5)}$=2.632, *p*=0.0464; CA1, $t_{(5)}$=2.807, *p*=0.0377; Sub, $t_{(5)}$=0.175, *p*=0.8679.

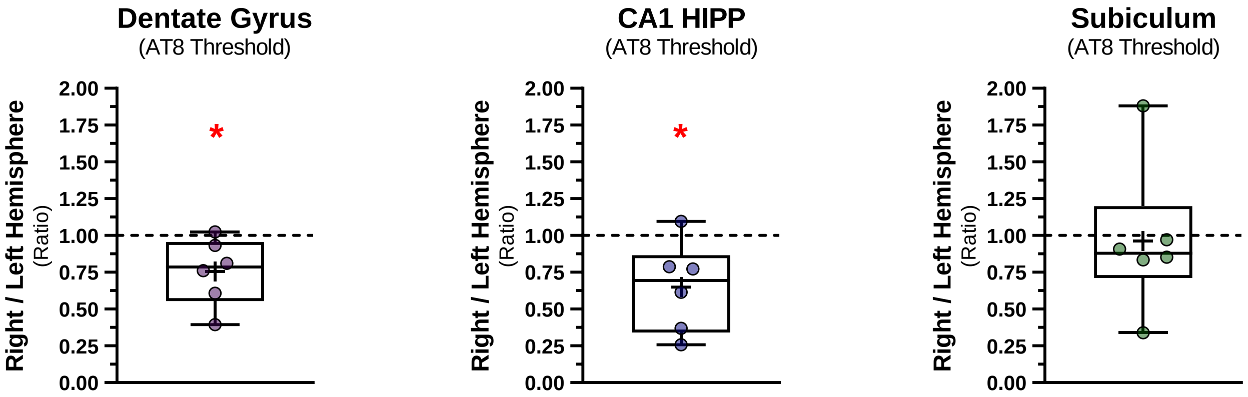

3. In figure 6, 7 and S5, the authors present sample photographs of stained slices. However, for the ordinary reader, it is almost impossible to detect the differences, especially between 'subtle reduction' and 'severe reduction' (figure legend fig. 6). They should also include magnified panels demonstrating the differences unambiguously.

We now provide higher resolution images of CP27+, AT8+ and MC1+ immunostained brain sections from two EC-Tau/hAPP mice in our chronic hM4Di DREADDs activation study, along with magnified 20X panels from three hippocampal subregions where we quantified tau marker immunoreactivity (Figure 5).

Regarding the difficulty in discerning differences in pathology from our former images and data presented, we agree with our reviewers and have made an important change to this revised manuscript. We wish to maintain our manuscript's focus on neuronal activity-mediated effects on pathological accumulation of Aβ and tau in the hippocampus. Thus, we restricted the presentation of our immunohistochemistry and tau marker quantification to EC-Tau/hAPP mice and EC-Tau mice that showed progression of MC1+ immunostaining in the DG and CA1 (n=8 mice, total). The mice presented in Figure 5 all showed reduced neuronal spiking and network activity after osmotic minipump implantation, indicative of hM4Di DREADDs activation *in vivo*. EC-Tau/hAPP mice that did not exhibit MC1+ immunoreactivity within the hippocampus, referred to as mice exhibiting "Early Tau" pathology in our former manuscript, were removed from the manuscript. Chronic hM4Di DREADDs activation appeared to reduce tau marker immunoreactivity in these mice as well, but shifted the focus away from the hippocampus to the entorhinal cortex. We now believe this complicated the data presented in our paper, an opinion shared by several reviewers.

4. The authors state, as their main point, that the attenuation of hyperactivity reduces Aβ and tau pathology (see title). This point needs further and independent substantiation through additional experiments. In fact, the authors did not compare the neuronal activity of treated hAPP mice with untreated WT mice to validate the attenuation. More importantly, Rodriguez et al. show that Gi-DREADD activation decreases neuronal activity in all tested genotypes. The fact that the reduction of neuronal activity was effective in EC-Tau mice (Fig. 6, 7) which do not have neuronal hyperactivity in the first place (Fig. 2), contradicts the authors' claim that the manipulation effectively attenuates neuronal hyperactivity. Finally, they need to repeat the experiments by using alternative and more specific ways of reducing the hyperactivity, perhaps by using optogenetics and/or by treating animals with low doses of benzodiazepines.

1.) We can agree that activation of hM4Di DREADDs in neurons leads to decreased spiking activity in all mice, which is consistent with our data demonstrating an effect in mouse models exhibiting hyperactivity (EC-Tau/hAPP and hAPP) and those that do not (EC-Tau and Non-transgenic control). While we disagree with the statement regarding contradictory evidence in EC-Tau mice, perhaps the reviewer is simply asking for specificity in reporting of electrophysiological data after DREADDs activation. If so, we can agree that it may be more appropriate to refer to chemogenetic manipulation as "reducing neuronal activity" and not just "hyperactivity", as it clearly drops activity in EC-Tau mice in addition to EC-Tau/hAPP mice. The overall effect on pathology that we found after DREADDs-mediated activity reduction is also supported by previous research showing the opposite effect, that heightened activity increases Aβ and tau pathology.

The authors have agreed to revise the manuscript title to reflect this specificity. The new manuscript title is, "Chemogenetic attenuation of neuronal activity in the entorhinal cortex reduces Aβ and tau pathology in the hippocampus".

2.) Neuronal activity after implantation of CNO-filled osmotic minipumps was always compared to individual activity measures prior to surgery. This within-subjects design allowed us to establish individual mouse baselines that we could then normalize the post-CNO neuronal spike counts to, which can vary greatly across mice. This is how we show acute (Supplemental Figure 3) and chronic (Figure 4A-B) DREADDs activation in our mouse models.

3.) We do not want to overreach with our conclusions. Thus, we do not conclude that AD can be treated by simply dampening neuronal activity. We speculate that attenuating aberrant neuronal activity can be a powerful tool if paired with other therapies that alleviate Aβ and tau aggregation (Page 12, Lines 452-454). Also, we state that additional studies will be required to determine if chemogenetic attenuation of neuronal activity in AD mouse models will have a positive effect on cognitive behavior (Page 12, Lines 463-465), which is an important step in validating an approach for treatment.

Finally, they need to repeat the experiments by using alternative and more specific ways of reducing the hyperactivity, perhaps by using optogenetics and/or by treating animals with low doses of benzodiazepines.

4.) Non-invasive, neuromodulatory approaches may one day offer therapeutic value to individuals exhibiting cognitive symptoms related to early AD (see Li-Huei Tsai's work in this field). However, we do not fully understand the impact of neuromodulation on Aβ and tau pathology in the AD brain or the mechanisms underlying AD pathology-associated neuronal network dysfunction. Thus, a major goal of our research program is to ameliorate neuronal network dysfunction in mouse models of AD pathology.

We chose to use chemogenetics (hM4Di DREADDs) in this study for several reasons, which we feel has advantages over alternative approaches like optogenetics. Chemogenetics is ideal for chronic neuromodulation, especially when a ligand is delivered via indwelling osmotic minipump, as we use in this study. This allows for a regulated release of the drug and circumvents the need for invasive daily CNO injections, which has been used in the past (Wu et al (2016); Yuan and Grutzendler (2016), Schultz et al (2018)). It also circumvents the problem of heat created by programmed light pulses needed for chronic optogenetic studies, which we worry would result in confounding effects unrelated to Aβ and/or tau pathologies. Selective targeting of neuronal cell types via DREADDS (CaMKIIa expressing neurons) also allows us to more tightly regulate the activity of neurons than drugs like benzodiazepines would. Additionally, pharmacological studies would require a significant increase in mouse numbers, as we would need an independent control group treated and not able to use a contralateral brain hemisphere.

Several cited studies report findings similar to our results and utilize different mouse models and technical approaches. We list some below with the main findings and mouse models used.

Increasing neuronal activity increases Aβ pathology
Yamamoto *et al* (2015) *Cell Reports*
Result: Five months of chronic optogenetic activation of the performant path increased Aβ release and deposition in the downstream dentate gyrus (outer molecular layer).
Mouse line: APP 695 transgenic mice (A7 line)

Modulating cortical activity can increase or decrease Aβ pathology
Yuan and Grutzendler (2016) *Journal of Neuroscience*
Result: 30 days of chronic chemogenetic DREADDs activation increased (hM3Dq) or decreased (hM4Di) Aβ pathology in downstream, projection regions of the brain.
Mouse line: 5XFAD transgenic mouse

Stimulating neuronal activity increases tau pathology
Wu *et al* (2016) *Nature Neuroscience*
Result: 20 days of optogenetic stimulation increases tau pathology in the hippocampus compared to non-stimulated, contralateral control hemisphere. 6 weeks of chronic hM3Dq DREADDs increased tau pathology in the entorhinal cortex.
Mouse line: rTg4510 transgenic mice

We have tried several attempts to independently substantiate our findings using alternative methods and mouse models of AD pathology. Unfortunately, technical difficulties prevented us from drawing conclusions from these additional studies. One such study is described briefly below, with some immunostaining figures shown.

Chronic levetiracetam treatment in EC-APP/Tau mice: We attempted to replicate out initial findings in an alternative mouse model (EC-APP/Tau mouse line; Khan *et al*, 2014. *Nature Neuroscience*) and with an alternative treatment strategy. Based on findings by Palop, Mucke and colleagues, we chronically implanted 10-month old EC-APP/Tau mice with osmotic minipumps (6-weeks) set to deliver 200mg/kg levetiracetam i.p. We also implanted 16-channel microdrives into the EC of these mice to record Baseline (pre- minipump) and Drug mediated changes in EC neuronal activity. Our conditions were: Saline, n=4; Levetiracetam, n=4. Unfortunately, three of our four Saline condition mice died within a week of their minipump surgeries, and while we allowed the remaining mice to finish their 6-week treatment plan, we could not accurately compare Aβ and tau pathological profiles in Levetiracetam treated mice to the one surviving Saline treated mouse. Electrophysiological results were promising (Lev appeared to reduce overall spiking activity) but not subjected to further analysis as this pilot was incomplete. Overall, we did not pursue a pharmacological approach to reducing neuronal activity because our focus was the EC and therefore we wanted to use a tool that would offer best regional specificity.

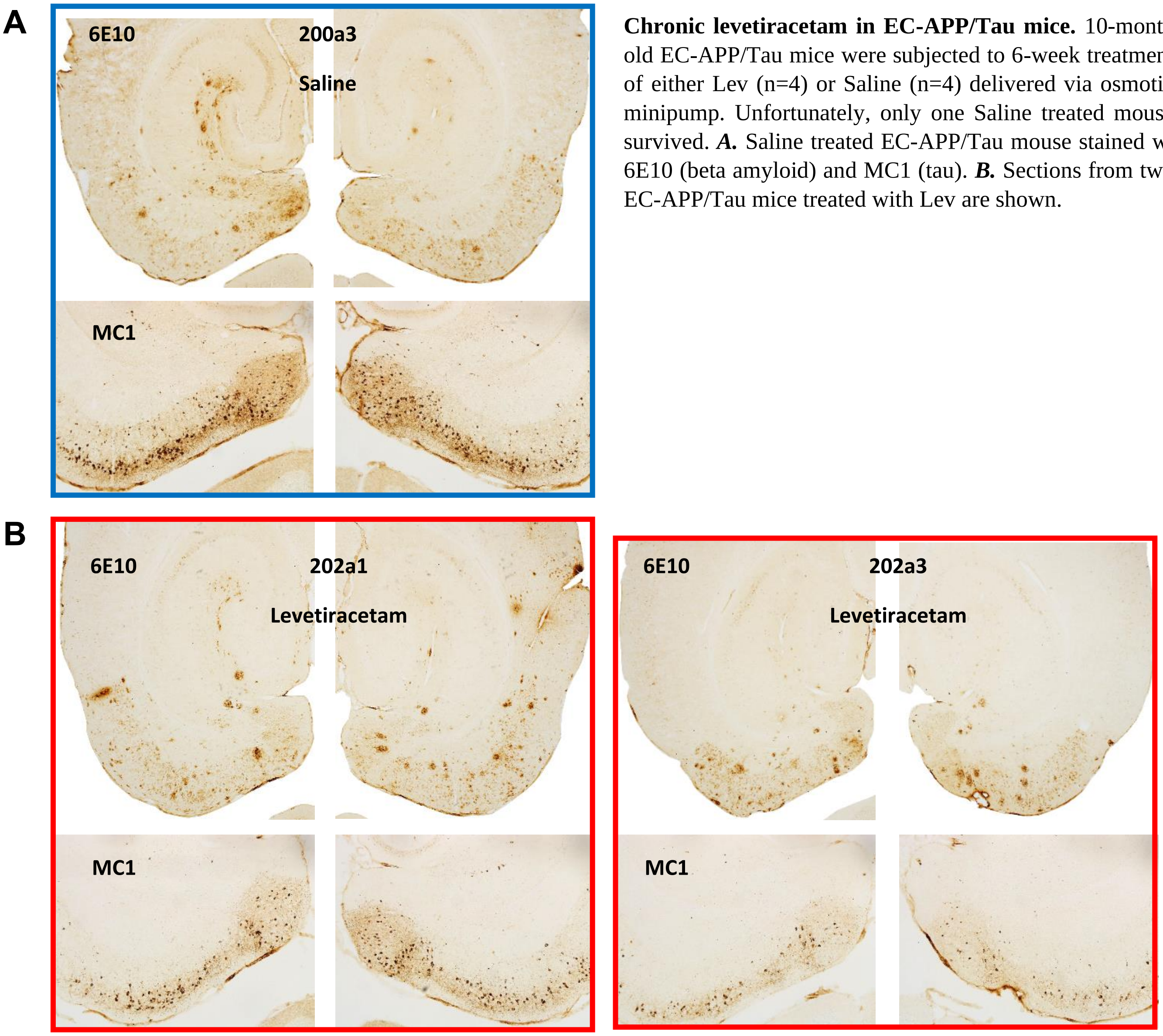

Chronic levetiracetam in EC-APP/Tau mice. 10-month old EC-APP/Tau mice were subjected to 6-week treatment of either Lev (n=4) or Saline (n=4) delivered via osmotic minipump. Unfortunately, only one Saline treated mouse survived. ***A.*** Saline treated EC-APP/Tau mouse stained w/ 6E10 (beta amyloid) and MC1 (tau). ***B.*** Sections from two EC-APP/Tau mice treated with Lev are shown.

Minor

1. Figure 4 does not provide any important information for the rest of the story and should be moved into the supplementary material.

> We have now moved the behavioral data to the supplementary material as Supplemental Figure 2 and have made changes suggested by the other reviewers. For instance, please see Reviewer #3's comments on potential genotype differences in time spent in the center versus outer edges of the arena. We analyzed our positional data and did not detect genotype differences in several measures.

2. In supplementary figure 3B, there is a callout to main figure 4G-H. This should be 5 G-H.

> We have corrected this mistake in the revised manuscript. Notably, left versus right hemisphere ROI analysis now appears as Supplemental Figure 4 and there are no callouts to the main figures.

Reviewer #2

In their manuscript, Rodriguez and colleagues use in vivo electrophysiological recordings as well as immunocytochemistry to characterize pathological network activity in the entorhinal cortex (EC) of mice overexpressing human mutant tau alone or together with the mutant amyloid precursor protein (APP). In line with recent in vivo observations in the parietal and frontal cortex, they identify amyloid, and not tau, as the major trigger for neuronal hyperactivity and impaired theta rhythmicity in the EC. DREADD-mediated reduction of neuronal activity of the EC reduced amyloid and tau pathology, suggesting an important role for hyperactivity in the propagation of the amyloid and tau burden and thus the overall disease pathology. These data strengthen the connection between pathological activity patterns and disease progression and suggest the reduction of neuronal hyperactivity as a prospective treatment strategy for reducing amyloid and tau pathology in humans. However, the following questions have to be addressed before the manuscript can be recommended for publication:

Major points:

1. The authors should improve presentation and quantification of data documenting the effect of DREADDs on tau pathology, as this is one of their major findings. Besides showing representative images, numbers of Tau-positive cells, identified by each of the three antibodies used, have to be presented (alike Fig. 1E).

> We thank the reviewer for their helpful critiques of our tau immunostaining figures in the previous version of our manuscript. All reviewers noted that the data presented required increased clarity and elimination of pseudo-replication in the semi-quantitative IHC analysis. We are happy to report that we have simplified the data presentation from our DREADDs-activated EC-Tau/hAPP mice (Figure 5). We now provide higher resolution images of CP27+, AT8+ and MC1+ immunostained brain sections from two EC-Tau/hAPP mice in our chronic hM4Di DREADDs activation study, along with magnified 20X panels from three hippocampal subregions where we quantified tau marker immunoreactivity (Figure 5). We also removed EC-Tau mice from our sample to eliminate mixing of genotypes into our analysis.
>
> In addition to the images shown, semi-quantitative threshold analysis was performed on sections from our experimental mice that captures pathological tau accumulation in the hippocampus (see methods and Supplemental Figure 4D-G). Data values are now shown for each mouse and for each marker in three hippocampal subregions (DG, CA1 and Subiculum), eliminating pseudo-replication from this figure (updated Figure 5).

2. The results presented in Figure 8 are hard to interpret, especially because the corresponding figure legend is unstructured and incomplete. In addition, it would be helpful to present images acquired at a higher resolution than those shown in Figure 6, 7 and Supplementary figure 8, so that the reader can understand which structures are labeled.

> Former Figure 8B & D were unfortunately missing a y-axis and the legend was an outdated version for the figure. We apologize for this oversight. We are happy to report that we have revised and simplified our tau immunostaining results following chronic hM4Di EC DREADDs activation, and have included new data from additional EC-Tau/hAPP mice in updated Figure 5 (n=6 EC-Tau/hAPP mice).
>
> Higher resolution images are now shown for two EC-Tau/hAPP mice in our chronic hM4Di DREADDs activation study. Color coded arrows (overlays) point to three hippocampal subregions chosen for tau IHC analysis.

3. Supplementary Figure 2: Total spike counts are reduced about 20% upon acute i.p. injection of saline. First, the reason for this effect remains unclear and is not discussed in the manuscript. Second, this effect size is similar to the 20% reduction of spike counts the authors detect after a 6-week-long treatment with CNO (Figure 5A), shedding some doubts about the efficiency of the chronic CNO treatment. Furthermore, the reduced theta rhythm that the authors describe in hAPP and ECTau/hAPP mice is not reverted by DREADD activation, but is further

decreased, pointing to a possible side effect of this treatment. This result has to be stressed and discussed in the manuscript.

The reviewer brings up a several great points which we address below. To begin, we feel that it's important to describe why and how the line graph in Suppl Fig 2A was generated. The purpose of our acute DREADDs activation studies (Suppl Fig 2) was to find a dependent measure that we could use to gauge whether our DREADDs were activated chronically via CNO delivered by minipump. Initial CNO experiments were then performed over three sessions in two mice (hM3Dq-expressing, n=1; hM4Di-expressing, n=1). The activity across four tetrodes were then normalized to their baseline conditions and averaged for each condition per mouse.

1.) We initially felt that showing the data from one mouse per condition would be acceptable if the data reflected three repeated experiments per mouse. However, we agree that it would be best to show activity changes across multiple animals, and not multiple experiments per mouse. Thus, we have updated this figure to show data from additional DREADDs-activated mice (n=6 mice total) in what is now Supplemental Figure 3A. The updated time-course data is also included below for reference, along with the statistical report (Two-way ANOVA with repeated measures, including Greenhouse-Geisser correction).

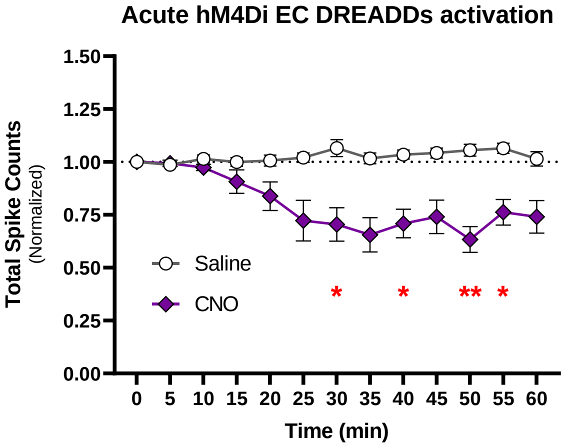

Two-way ANOVA w/ repeated measures

Interaction $F_{(11,110)}$ = 5.448, $p < 0.001$
Time $F_{(2.4,23.5)}$ = 3.112, $p > 0.05$
Drug $F_{(1,10)}$ = 30.490, $p < 0.001$

Sidak's multiple comparisons test

CNO vs Control, 30 min; $p < 0.05$
CNO vs Control, 40 min; $p < 0.05$
CNO vs Control, 50 min; $p < 0.01$
CNO vs Control, 45 min; $p < 0.05$

2.) We initially hypothesized that the ~20% reduction in spike counts after Saline conditions reflected low levels of locomotor activity and perhaps increased sleeping in the mouse shown in our original manuscript submission (ID: 302805a5). However, as we collected more data in new mice, we did not see this same pattern of activity repeated. Instead we found that the variability in the activity changes across tetrodes was high after Saline for Mouse 302805a5. Thus, we conclude that the reduction in activity we originally showed was due to erroneously averaging data across tetrodes in several experiments and not across individual mice, which would minimize the variability in activity patterns across tetrodes. We have corrected this and show new data with multiple hM4Di EC DREADDs expressing mice (Supplemental Figure 3A and above), and show the individual mouse traces depicting neuronal activity after Saline and CNO injections below. These graphs include data from Mouse 302805a5.

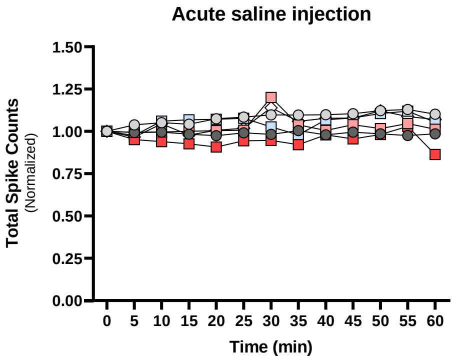

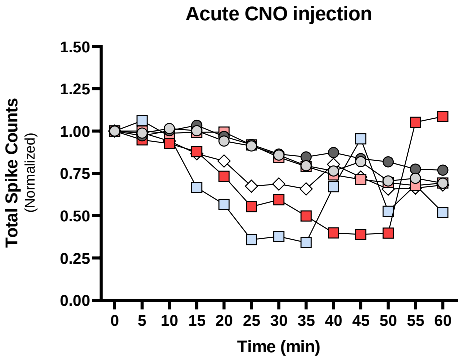

3.) We thank the reviewer for commenting on the decrease in % Theta power following chronic hM4Di DREADDs activation with CNO. We agree that this reduction in % Theta power is not necessarily a desirable effect. For our purposes, this measure was used to track hM4Di DREADDs activation *in vivo*, along with changes in neuronal spike counts normalized to individual mouse 'Baseline' measures. Ideally, we would want to find an appropriate level of neuronal attenuation that would bring % Theta power and neuronal firing rates to non-transgenic Control mouse levels. This point is stressed in this revised manuscript (Page 11, Lines 429-433).

In order to determine whether the further reduction in % Theta power observed after chronic DREADDs activation was due to effects on locomotor activity, we analyzed the mean speed (cm/sec) and total distance traveled (m) during the recording sessions post-CNO minipump implantation (see line graphs below). Since we did not find an effect of chronic DREADDs activation on these two behavioral measures, we could not conclude that reduced % theta power was due to locomotor effects.

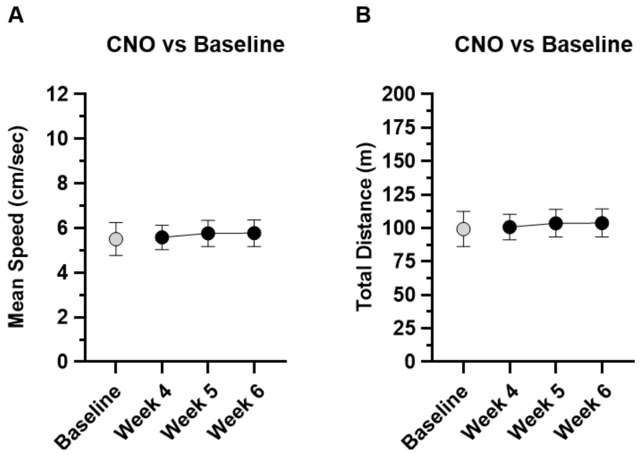

4.) The reduction in % Theta power reported after acute injection of CNO is now updated in Supplemental Figure 3C-D. Data from additional mice have been included to increase the sample size (n=8, total). For convenience, this data is also shown below.

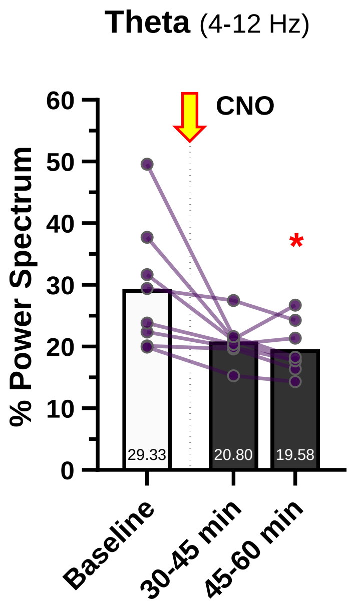

Repeated measures one-way ANOVA
$F_{(2,7)} = 7.079$, $p < 0.05$

Dunnett's multiple comparisons test
45-60 min vs Baseline, $p < 0.05$

4. For statistical comparisons, the authors repeatedly pool several different genotypes and groups (e.g. Figure 5G, 5H, Supplementary Figure 3C, 3D) without any explanation or justification. This is a very unconventional way

to evaluate data and should be avoided. Instead, the number of mice for each group should be increased to reasonable values (≥ 5 mice).

In this revised manuscript, we have increased the sample sizes for statistical comparisons in updated Figure 4E (n=9 mice, total) & Figure 5 (n=6 mice, total), which depict hM4Di DREADDs-mediated changes in hAPP/Aβ and tau pathology, respectively. Moreover, we have restricted analysis to DREADDs-activated EC-Tau/hAPP mice, eliminating analysis of mixed genotypes. In addition, we have simplified the figures by showing higher resolution, high magnification images of 6E10+, CP27+, AT8+ and MC1+ immunostaining in the hippocampus.

There are instances where the authors feel that it is justified to combine mice into the analysis from different genotypes. These are described here along with the justification for their inclusion…

1.) Figure 4: n=9 mice assigned to Control conditions (described in Methods, Pages 17-18, Lines 653-657). These mice were used to examine side-to-side variability in 6E10+ immunoreactivity under conditions which were not hypothesized to induce changes in pathology. The paired *t* test shows that there is no aberrant laterality in 6E10+ staining inherent across hAPP/Aβ overexpressing mice.

2.) Supplemental Figure 3A & D: n=6-8 mice total used to examine changes in EC neuronal activity after Saline or CNO injections. The purpose of these experiments was to qualify the metrics used to track *in vivo* hM4Di DREADDs activation in our chronically treated mice. At 16-months of age, the degree to which neuronal activity and % Theta power are reduced following DREADDs activation is likely more dependent on viral delivery into the EC and CNO injection rather than genotype.

3.) Figure 4A-B: n=12 mice total whose data demonstrate that reductions in neuronal activity and % Theta power can be tracked *in vivo* following CNO-minipump implantation. Our justification for including this data follows our reasoning for including acute CNO injection data from combined mice in Supplemental Figure 3. We do not see evidence for genotype-mediated differences in CNO activation in our mice at 16-months of age. Rather, the degree of DREADDs activation is more likely due to viral delivery of DREADDs into EC, DREADDs expression in EC neuronal populations and degree of CNO (or converted clozapine) binding to DREADDs than genotype.

5. Because the LFP experiments presented in Figure 3 were performed in the light phase (p. 15, line 537), it is expected that sleep states represent a considerable fraction of the recording period. Indeed, eliminating LFP activity that occurred during "bouts of immobility" altered the results obtained (Supplementary Figure 1). In addition, it is well known that sleep architecture and the duration spent in the awake/sleep state is altered in different AD mouse models. As it stays now, the composition of the recording period in terms of wake and sleep states, the duration of the respective states and their relative contribution to the recorded signals in the different mouse strains used in this study remains unclear. Without reliable brain state segregation the data are very difficult to interpret.

Sleep states do not represent any fraction of our recording experiments. All recording sessions are 30 min in duration only and are conducted during free exploration of an arena with various visual cues adding in navigation. Updated trajectory maps showing the total distance traveled during a typical recording session are shown in Supplemental Figure 3, along with behavioral data. In our experiments, mice actively explore and demonstrate foraging behavior during this recording period, and we do not observe sleeping. Thigmotaxic behavior is observed, and occasionally mice will spend time in the corners of the arena. Speed filtering is performed on the electrophysiology data because it has been shown that running speed can influence theta oscillations in the region, but not because we are trying to eliminate sleep state activity.

6. Figure 6: Why do the EC-Tau and EC-Tau/hAPP animals of the same age (16 months) show so different levels of pathology? As both female and male mice were used, could this be a gender effect? If mice have to be separated into two groups (Early vs. Advanced pathology) the numbers of experimental animals in each group have to be increased (now: ~ 2 animals per group).

Reviewer #2 brings up an important issue with the tau immunohistochemistry data presentation in our original manuscript. The confusion of the 'Early' and 'Advanced' tau pathology was noted by all reviewers, and thus we have made significant changes to the data presentation in this revised manuscript to clarify the results that we initially intended to show. We provide our explanation of the variability in tau pathology seen in our transgenic line, which is also found in the responses to critiques from our other reviewers.

We are happy to report that we have revised and simplified our tau immunostaining results following chronic hM4Di DREADDs activation, and have included new data from additional EC-Tau/hAPP mice in updated Figure 5 (n=6 EC-Tau/hAPP mice). We have also removed EC-Tau mice from the analysis, eliminating data pooling in the immunohistochemistry quantification. Also, former Figure 8B & D were missing a y-axis and the legend was an outdated version for the figure. This has been corrected in the updated Figure 5. As for dividing the data by sex, we refer the reviewer to Appendix 1, where we perform our analyses using sex as an independent variable. Unfortunately, dividing the tau IHC data by sex reduces the power of our analyses to a point where we are not able to make firm conclusions on sex differences.

1.) The 16-month timepoint was chosen for our experiments on the basis that pathological tau (MC1+) had begun appearing in hippocampal subregions, suggesting propagation from a primary point of transgene expression (entorhinal cortex) into synaptically connected, downstream brain regions. However, we observed some variability in the levels of tau pathology in both EC-Tau and EC-Tau/hAPP mice at the 16-month timepoint, which is why we see varying levels of aggregated tau in mice from the same strain (e.g. EC-Tau/hAPP mice, former Figure 6F versus P, etc.). In the previous manuscript, we wanted to show as much of the histology as possible to illustrate the effects of DREADDs on tau, as the DREADDs-associated reduction in tau immunostaining was quite striking in some mice (former Fig 6F) but somewhat subtle in others (former Fig 6A).

We realize that focusing on DREADDs-mediated reductions in tau pathology resulted in a confusing presentation of the data where extreme cases of variability in tau pathology were shown. We do not feel this is representative of the effects we were trying to show. In general, we observed that EC-Tau/hAPP mice as a group had significantly increased tau pathology along the EC-HIPP network than age-matched, littermate EC-Tau mice. Data described in Figure 1 of our manuscript agrees with previously published data from our lab describing an effect of amyloid pathology on tau propagation in a similar mouse model (Khan *et al*., (2014) *Nature Neuroscience*. Figure 6C). Our data also agrees with data reported in a similar mouse line from Dr. Bradley Hyman's group (rTgTauEC x APP/PS1; Pooler *et al*., (2015) *Acta Neuropathologica Communications*. Figure 2). Figure 2 from Pooler *et al*., (2015) is shown below to illustrate the similarity in these results from different lab groups. Finally, we felt that within-subject comparisons of Aβ and tau immunoreactivity across hemispheres was the best way to test our hypotheses, which factor in inherent variability in pathology.

Pooler *et al* (2015) *Acta Neuropathologica Communications*

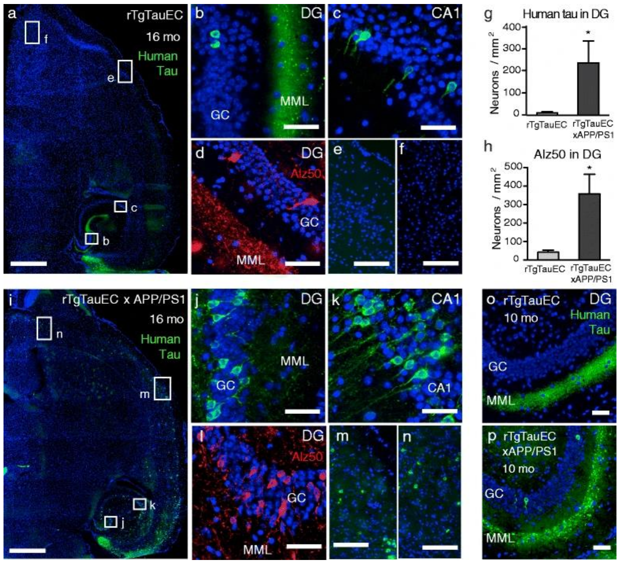

Figure 2. Propagation of tauopathy along neural circuits is exacerbated by amyloid pathology

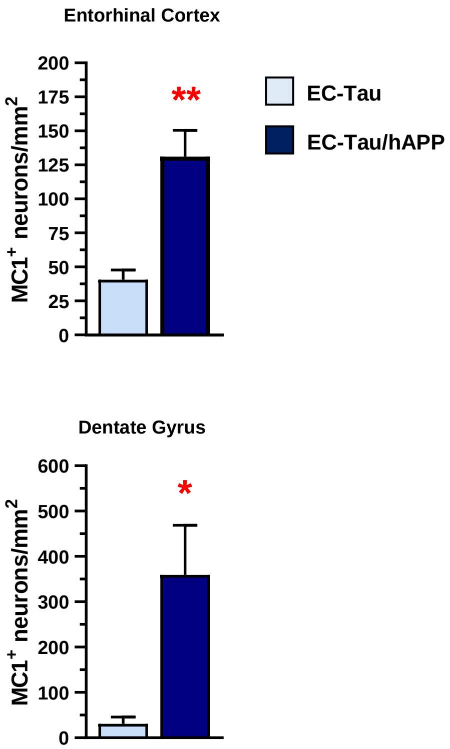

Regarding the difficulty in discerning differences in pathology from our former images and data presented, we agree with our reviewers and have made an important change to this revised manuscript. We wish to maintain our manuscript's focus on neuronal activity-mediated effects on pathological accumulation of Aβ and tau in the hippocampus. Thus, we restricted the presentation of our immunohistochemistry and tau marker quantification to EC-Tau/hAPP mice and EC-Tau mice that showed progression of MC1+ immunostaining in the DG and CA1 (n=8 mice, total). The mice presented in Figure 5 all showed reduced neuronal spiking and network activity after osmotic minipump implantation, indicative of hM4Di DREADDs activation *in vivo*. EC-Tau/hAPP mice that did not exhibit MC1+ immunoreactivity within the hippocampus, referred to as mice exhibiting "Early Tau" pathology in our former manuscript, were removed from the manuscript. Chronic hM4Di DREADDs activation appeared to reduce tau marker immunoreactivity in these mice as well, but necessarily shifted the focus away from the hippocampus to the entorhinal cortex. We now believe this complicated the data presented in our paper, an opinion shared by several reviewers.

Other points:

1. Figure 2 C: In the figure legend, the authors state that EC-Tau/hAPP and hAPP are significantly different from control. However, it seems to be rather clear from the graph that the largest difference was observed between EC-Tau and control mice. This should be mentioned and discussed.

Cumulative frequency distributions of the average neuronal firing rates (Figure 2B) and inter-spike intervals (Figure 2C) have been updated in this revised manuscript, which now includes new data from n=11 additional mice to make up the following sample sizes: Control, n=8 mice (n=386 single-units total); EC-Tau, n=7 mice (n=404 single-units total); hAPP, n=8 (n=532 single-units total); EC-Tau/hAPP, n=8 (n=588 single-units total). Updated Figure 2C shows the largest group differences in median ISIs between EC-Tau/hAPP mice versus Control mice, and hAPP mice versus Control mice.

2. There seems to be a clear discrepancy between the results shown in Figure 2B and Figure 2C. Whereas the

Average frequency rate is clearly shifted to higher values in hAPP and EC-Tau/hAPP mice compared to EC-Tau and control mice, the Median ISI did not show any obvious differences between hAPP / EC-Tau/hAPP and control / EC-Tau mice, as would be expected from alterations in the frequency rate. The authors need to clarify and discuss this issue.

We thank the reviewer for pointing this out. Please see our previous comment, where we report that updated Figures 2B-C show the corrected cumulative frequency distributions that match average firing rate data and ISI data.

3. In all figures, the significant differences between each pair of experimental groups (as indicated by post hoc tests) should clearly be indicated by lines and asterisks, not just the significance of the Kruskal-Wallis test or ANOVA.

This is a great suggestion which will believe will provide clarity for our readers. We now provide lines and asterisks to indicate group differences following *post hoc* analyses in data figures throughout the revised manuscript.

4. Especially because the behavior/the state of disease progression of individual mice is very heterogeneous (as pointed out by the authors several times in the text), the results obtained from all measured neurons should not simply be pooled together (see for example Figure 2B and 2C). The authors should rather calculate median/average values for each parameter in each mouse and then compare these values between different groups.

We thank Reviewer #2 for raising this issue. We are happy to announce that we have now included new data from additional mice to our electrophysiology datasets and have updated our statistical analyses to include individual values per mouse, eliminating data pooling. An updated Supplemental Table 1 containing Mouse IDs, Sex, AVG Firing Rates and Bursting % per genotype is included.

5. Line 222: “Single, high dose CNO (5 and 10mg/kg, i.p.) injections reliably induced DREADD expression”…. CNO is not inducing DREADD expression, isn’t it?

We thank the reviewer for the oversight. We have now changed the sentence to read, “Single, high dose CNO (5 and 10mg/kg, i.p.) injections reliably altered EC neuronal activity and theta power in hM4Di EC DREADDs-expressing EC-Tau mice and hAPP mice (Supplemental Figure 2)…”. Page 7, Line 223.

6. Line 246: “Decreased 6E10+ immunoreactivity was evident throughout the DG, CA3, and CA1 strata (right HIPP: 8.85 ±1.03 vs left HIPP: 14.92 ± 1.95 %)”. What do the numbers in brackets represent?

In our original manuscript, the numbers in brackets represented the averaged % of 6E10 immunoreactivity that appears in our hippocampal ROI above threshold for n=7 mice analyzed. However, in our revised manuscript, we have removed these numbers from the manuscript body and instead report mean values for Aβ Coverage (%) in hippocampus at the bottom of the bar graphs (Figure 4D-E).

Reviewer #3

In this potentially interesting paper, Rodriguez et al initially demonstrated, as previously reported, that crossing an APP transgenic mouse with mutations causing plaque deposition with a mouse transgenically overexpressing mutant Tau which develops neurofibrillary tangles results in an increase in the formation of neurofibrillary tangles. In particular in this case, use of a mouse in which the Tau transgene is largely restricted to the entorhinal cortex, they report a dramatic increase of the spread of tangles into the hippocampus.

They then go on with electrophysiological recordings in vivo in behaving mice to demonstrate that, as reported previously that transgenic expression of mutant APP together with plaque deposition results in increased activity particularly in excitatory neurones and possibly selectively affecting theta and gamma frequencies. They report that combining the two transgenes has little effect on this increase and the network changes have no effect on locomotor activity in an open field. In order to assess whether the increased spread of Tau pathology may be due to the increased activity in the APP transgenic mice, they then manipulate activity in one hemisphere using DREADS; comparing the other hemisphere of the same mouse in each case. They report that decreasing activity decreases the 6E10 staining (~deposition of amyloid plaques) and also the Tau staining using a range of antibodies. Although many of the reported findings in this paper are not entirely novel the experimental approaches used are elegant and the possibility of comparing some of the data and correlating between effects in individual mice would be interesting to explore. Unfortunately, although it sounds impressive when the total number of mice used is quoted, the sample size per group is often extremely small (usually n=4) and the method of analysis using pseudo replication of the multiple cells within each mouse is problematic. The initial demonstration of increased spread of Tau pathology in the double transgenic mice compared to the single transgenic EC Tau is however convincing and interesting.

We thank Reviewer #3 for raising the issue of low n's per group and data pooling. We are happy to announce that we have now included new data from additional mice to our electrophysiology datasets and have updated our statistical analyses to include individual values per mouse. For instance, in our single-unit analyses (Figure 2 & Supplemental Figure 1), we included additional data from 11 mice total to generate the following sample sizes: Control, n=8 mice (n=386 single-units total); EC-Tau, n=7 mice (n=404 single-units total); hAPP, n=8 (n=532 single-units total); EC-Tau/hAPP, n=8 (n=588 single-units total).

An updated Supplemental Table 1 containing Mouse IDs, Sex, AVG Firing Rates and Bursting % per genotype is included.

The mice

The methods state that male and female mice were used but there is no indication of which data relate to which gender or if the genders are evenly distributed across groups. As there may be a difference between genders, especially in the aged mice, differences in genders between the genotypes used could influence the data and so this information is essential.

We apologize for the omission of sex in our original manuscript. We are happy to report that we now provide the sex of experimental mice in this revised manuscript, which includes new data from additional mice. Sex is reported in all Figure Legends and methods section, as well as Supplemental Table 1 matched to Animal IDs for single-unit electrophysiology.

While we have increased the overall number of animals in this revised manuscript to make firm conclusions that support our claims, dividing the data by sex reduces the power of our analysis to a point where we are not able to make conclusions on sex differences at this point. However, please see Appendix 1 for a report on the breakdown of our electrophysiology and immunohistochemistry data by sex and statistical analyses which shows interesting differences.

Electrophysiology

The electrophysiological data shown in Figure 2 is problematic. Very likely the overall conclusion are mostly correct, although a little overblown, however to reach these conclusions the data should be analysed per mouse not using pooling of all cells of a genotype irrespective of the mouse in which is was recorded. Table 1 gives the individual data for much of Fig 2 and so the difference in the outcomes can be calculated using the data from each mouse. I have thus concentrated in detail on this point for Fig.2 but the problem applies to the electrophysiology throughout the paper. The data in the Table for experiments in Fig.2 are replotted in this way in the Appendix attached. The overall firing rate comes out with very similar results but, although not vastly different, the statistics change somewhat for the wide and narrow spiking cells. The problem can be particularly seen for example in the double transgenics Wide spiking neurones where one mouse with the highest mean frequency of firing (~8Hz) contributes 101 cells (representing almost half of the cells sampled) while another with cells averaging at about 2Hz contributes only 11 cells. Clearly if the cells are all pooled the average of the first mouse will greatly weight the result which is not appropriate. It is possible that the difference in average spiking relates to the position of the electrodes or the stress of that particular mouse or one of many other issues meaning that the recordings within one mouse, even if very varied are not independent of each other. Moreover the reason for managing to record so many ore cells in this mouse is not clear. Is it easier to find recordings in rapidly spiking cells biasing the results in that direction, or are more neurones surviving in this animal or is the animal very inactive making the recordings are more stable? There are many possibilities. Analysing per mouse shows up this variability between animals and results in no significant differences for narrow spiking cells between genotypes and a main effect for APP ($p<0.01$) for wide spiking with no interaction with the Tau transgene. To say anything much about the double transgenics, the number of mice tested would have to be considerably increased. This problems runs throughout the electrophysiological experiments but the average data per mouse is not given for other experiments and so it is impossible to assess the extent to which the outcomes would change. However all data should be analysed per mouse and not pooled in mice with massively different sample sizes. The n for analysis is the n for number of mice not for number of cells recorded.

We thank the Reviewer for bringing to light these issues with our electrophysiology data. We especially want to thank them for taking the time to re-analyze our results from the table data provided, which shows an attention to detail that we admire. Critique responses appear below.

1.) This revised manuscript now includes new electrophysiology data from additional mice (Figure 2 and Supplemental Figure 1; please see Supplemental Table 1 for breakdown) and statistical analyses are now performed on an individual mouse basis.

2.) Mouse ID: 291117a2, EC-Tau/hAPP (Male). An examination of our recording records showed that this mouse was implanted with a 32-channel microdrive, where single-unit data was collected across 8 tetrodes instead of 4 (16-channel). We collected a greater number of neurons from this mouse than any other, and the reviewer was right to point out that over-representation from this mouse might skew the results of our analysis.

To minimize any effect of oversampling from this mouse, we randomly selected n=74 single-units from Mouse 291117a2 after shuffling the dataset 50X. We arrived at 74 single-units by averaging the number of single-units collected from the rest of the mice in the EC-Tau/hAPP group (n=7 mice; 73.428 single-units collected on average). After examining the data (Figure 2B, E-F and included below for easy visualization), we see that reducing the number of single-units for this mouse did not significantly change the AVG FRs for either narrow spiking (NS) or wide spiking (WS) cells. We did not identify outliers in our dataset using the robust regression and outlier removal method (ROUT) in GraphPad Prism (Q=1%). We therefore wish to include the data from n=74 single-units from 291117a2.

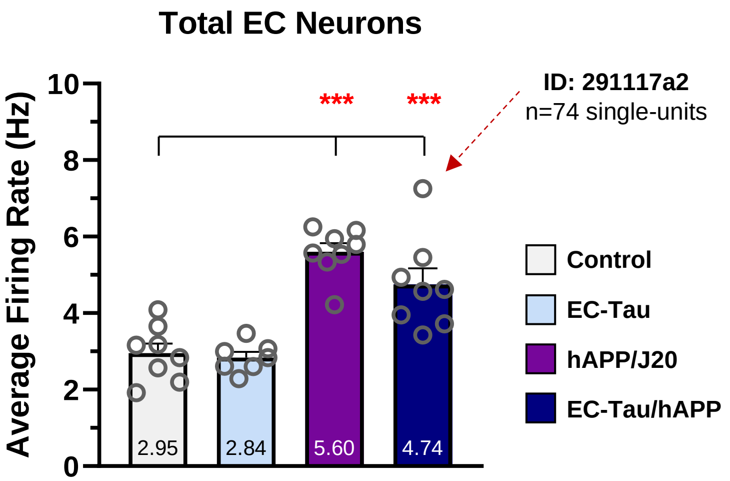

One-way ANOVA
$F_{(3,27)} = 21.80$, $p < 0.001$

Dunnett's multiple comparisons test
hAPP/J20 vs Control, $p < 0.001$
EC-Tau/hAPP vs Control, $p < 0.001$

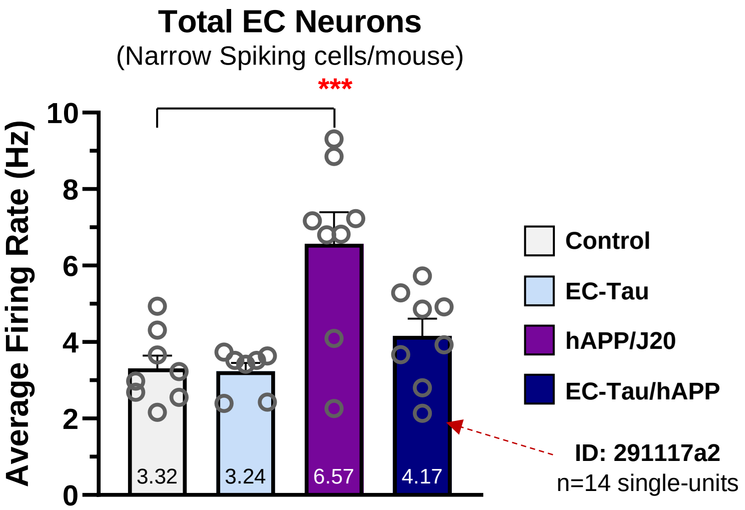

One-way ANOVA
$F_{(3,27)} = 8.90$, $p < 0.01$

Dunnett's multiple comparisons test
hAPP/J20 vs Control, $p < 0.001$

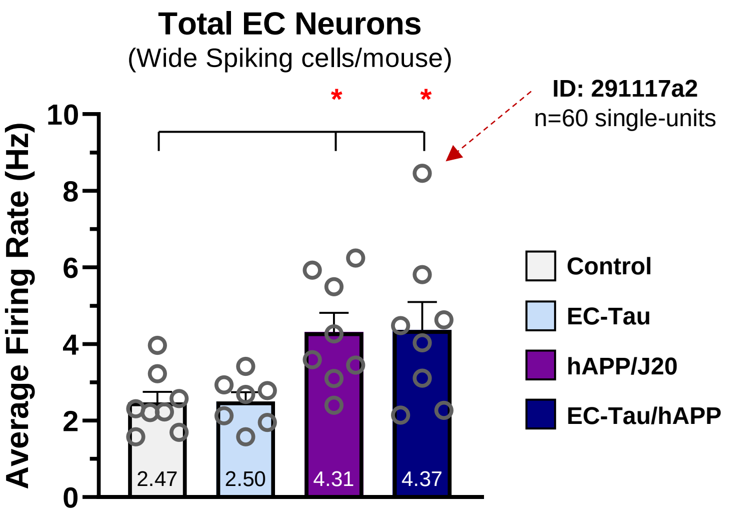

One-way ANOVA
$F_{(3,27)} = 4.71$, $p < 0.01$

Dunnett's multiple comparisons test
hAPP/J20 vs Control, $p < 0.05$
EC-Tau/hAPP vs Control, $p < 0.05$

Another question arises about the narrow and wide spiking neurones. It is not at all clear to me why they are divided in the way that they are using the equation: (Valley-1st Peak)/2 + 1st Peak
This just doesn't seem to make any sense and on looking at the paper referred to I believe it is a misunderstanding of how the spike width was measured in an individual spike in which the valleys refer to the minima before and after the peak of the voltage trace and not to a histogram of the pooled data. As far as I can see the division should just be at the valley or to be strictly correct, 2 Gaussians should be fitted and the number of events in each group should be the area under the curve of each Gaussian. If there is some reason why the above equation should be applied then it needs to be explained much more clearly.

This is a very important point and we thank the reviewer for bringing up the issue with the spike-width cutoff. We have now corrected our separation of Narrow-Spiking (NS) and Wide-Spiking (WS) neurons. As suggested by the reviewer, we split the two neuronal populations at the valley of the bimodal distribution once all of the new data from additional mice were included (1,910 single-units across 31 mice total). Please see revised Figure 2D for the updated cutoff (340us) and revised Figure 2E-F for average firing rates in NS and WS single-units. See also a revised description in the Methods (pgs. 551-555).

I am also not at all sure what panel D is supposed to add especially as there is no indication of which cells come from individual mice.

The scatter plot was initially added to show a higher proportion of single-unit average firing rates in hAPP mice and EC-Tau/hAPP mice (purple) compared to non-transgenic Controls and EC-Tau mice (light gray). We felt that this would allow our readers to easily see that hAPP/Aβ was associated with EC neuronal hyperactivity at the neuronal population level.

We decided to exclude this scatter plot in the revised manuscript because it did not sufficiently add information to the data presented in Figure 2. We want our readers, especially those that are not AD experts, to easily be able to draw conclusions from our data figures.

The problem of pseudoreplication runs through the analysis of theta rhythms etc (although how these are sampled at all is not entirely clear) and all the DREAD electrophysiology experiments and so it is very hard to comment on these experiments until they are correctly analysed. Note that here and wherever Analysis of Variance is used it would be more appropriate and possibly bring extra information to use a 2 way Analysis of Variance with APP genotype and Tau genotype as the two factors. However ideally multivariate analysis could be used taking into account to analyse the individual cell data, the identity of the mouse, the gender of the mouse etc.

We are happy to clarify for the reviewer that the LFP data was analyzed on a per mouse basis and that group averages were compared using a One-way ANOVA after testing for normality. In this revised manuscript, we have included LFP data from additional mice (n=8 new mice), which brings the sample sizes to the following: Control, n=9 mice; EC-Tau, n=8 mice; hAPP, n=10 mice; EC-Tau/hAPP, n=10 mice. Please note that generating LFP data (% of power spectrum values) using our automated *BatchPowerSpectrum* pipeline (described in Methods and freely available) is much easier than generating single-unit data, which is not automated and requires careful manual effort after automated spike sorting (also described in Methods). That is why there are more mice per genotype in revised Figure 3 than in revised Figure 2.

In Appendix 1, we report our electrophysiology and immunohistochemistry analyses (Figures 2-3, Figure 4-5 and Supplemental Figure 1) by sex.

Behaviour

Fig. 4. No change is seen in activity between the genotypes. However the sample traces have been chosen poorly in panel A as it appears as though there is more activity or a different pattern of activity for the hAPP and double

transgenics. Hence more typical traces of average rather than maximum activity should be chosen. It might also be interesting to analyse time spent on the edge versus in the centre of the arena which relates to anxiety. Increased anxiety has also been frequently shown to be a feature of transgenic APP mice.

We thank the reviewer for pointing out that the representative trajectories chosen for hAPP mice and EC-Tau/hAPP mice gives the impression that they have a different pattern of activity than Controls. In our study, we did not find sufficient statistical evidence for genotype differences in motivated foraging behavior in an open field.

In this revised manuscript, we have moved the updated behavioral figure to the supplementary materials (Supplemental Figure 2). We updated panel A to include different trajectories for Control and EC-Tau/hAPP mice, which we feel no longer leads our readers into thinking behavioral patterns are different across genotype in this particular study.

While we did not suspect group differences in time spent along the edges vs arena center from our initial overview of the trajectories, we nonetheless created an automated Quadrant Analysis tool in Python that allowed us to quantify time (sec), distance traveled (m) and % coverage in defined zones (4x4) within our behavioral arena. To illustrate, the example trajectory for one Control mouse - ID 60704a4 - appearing in Supplemental Figure 2A is shown (left) with the arena broken up into sixteen equal zones (right). Four zones in the center area or the arena make up the Inner Zones (RED) and the remaining twelve zones along the edges of the arena make up the Outer Zones (Blue). We performed a Two-way ANOVA using Arena Zone and Genotype as independent variables and followed up with Tukey's Multiple Comparisons test to look for cell mean differences. We did not find any significant effect of Genotype in our analysis, only a strong effect of Arena Zone (with no Interaction between factors). *Post hoc* comparisons revealed group differences in Inner versus Outer Zone comparisons only.

These data, along with those in Supplemental Figure 2, suggest that motivated foraging behavior in an open arena is not significantly affected by Aβ and tau pathology along the EC-HIPP network in 16-month old mice.

Mouse ID: 60704a4 Control

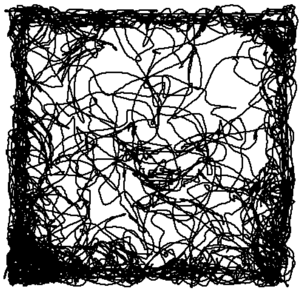

Arena area divided into 16 equally sized zones, allowing for comparisons of behavioral measures within Inner and Outer Zones across genotype.

Inner Zones = RED
Outer Zones = BLUE

Quadrant Analysis Map

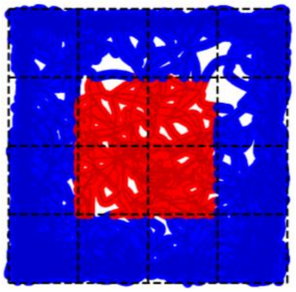

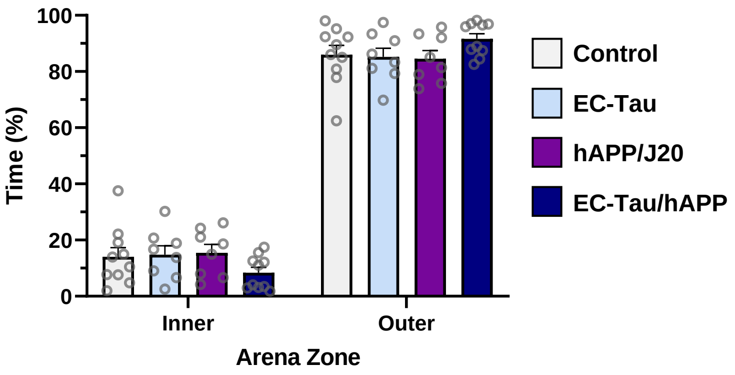

Two-way ANOVA

Interaction $F_{(3,64)} = 2.721$, $p > 0.05$
Arena Zone $F_{(1,64)} = 1327$, $p < 0.001$
Genotype $F_{(3,64)} = 1.399$, $p > 0.05$

Tukey's multiple comparisons test
Inner zone (time %) vs Outer zone (time%) across all genotypes.

No significant *post hoc* test differences in Inner Zones (time %) across genotype or Outer Zones (time %) across genotype.

Immunohistochemistry

On the whole the immunohistochemistry is pretty clear although some questions also arise here. In Figure 5G the % 6E10 above threshold decreased with the DREAD activation with eGFP control showing around 15% and the paired hemispheres with the DREADS at around 10%. The controls show no difference between hemispheres however, curiously, the levels are similar to the level in the treated hemispheres in the test animals. Hence in the untreated control shown in panel H both hemispheres are reported at around 10%. Hence it seems that the eGFP group (CNO in the absence of the DREAD) is increasing the level rather than the DREAD decreasing it. Is there any explanation for this?

In this revised manuscript, we have increased the sample size of EC-Tau/hAPP mice to n=9 in Figure 4 and have excluded the CNO-treated hAPP mice from the analysis, at our reviewers' request. We have also simplified the figure by showing high magnification images of 6E10+ immunoreactivity in the hippocampus (left & right hemispheres) from two CNO-treated EC-Tau/hAPP mice (Figure 4D). Since we added 5 additional EC-Tau/hAPP mice to the analysis (Figure 4E), and given the concerns Reviewer #3 shared regarding the levels of 6E10+ immunostaining in mice assigned to the control conditions, we reasoned that it was best to re-image all the tissue sections for this figure and process them for threshold analysis together. We suspected that the similarity in % levels of 6E10 in left hemisphere of mice in control conditions (former Fig 5H) and right hemisphere in EC-Tau/hAPP mice (former Figure 5G) was due to variability in image acquisition and processing, as the sections from mice in control conditions was performed all together first and then followed by the CNO 1mg/kg/day treated EC-Tau/hAPP mice at a later time.

Re-imaging all the 6E10 immunostained sections and processing all images together led to decreased variability in the % 6E10 levels reported (updated Figure 4E-F). In control conditions, Aβ coverage in left and right hemispheres of mice was 14.81% and 13.99%, respectively (not significantly different using a paired *t*-test). The remaining differences (range) in hippocampal Aβ coverage across mice are likely due to true variability in Aβ pathology in individual mice and not the imaging/analysis process. This is one of the motivating factors to designing our experiments for within-subject comparisons. In the Table below, we provide the Aβ Coverage % values for left and right hemispheres of n=5 mice in the control conditions that did not have AAV5-CaMKIIa-eGFP injected into their left EC. Notice that there is a range in Aβ coverage %, from ~13% to ~20%. Thus, we do not think that eGFP expression is driving variability in Aβ coverage % in our mice.

Mouse ID	Genotype	Sex	Left HIPP - Aβ Coverage %	Right HIPP - Aβ Coverage %
251117-1	EC-Tau/hAPP	F	20.53	19.23
251117-2	EC-Tau/hAPP	F	16.88	13.80
271031-2	hAPP	F	13.63	13.34
261031a3	hAPP	M	15.02	15.13
301031a2	hAPP	M	13.03	13.55

Importantly, while we report that 6E10+ immunoreactivity – and to an extent CP27+, AT8+ and MC1+ immunoreactivity - was decreased by chronic $hM4D_i$ DREADDs activation, we acknowledge that this alone will likely not be sufficient to alleviate symptomatology in human AD. We suggest that alleviating neuronal hyperactivity and network dysfunction in combination with immunotherapies that reduce aggregated Aβ and tau early in AD may provide an advantage over immunotherapies alone (Page 12, Lines, 452-454).

Fig 6 also raises some questions. It is possible to see a difference between the treated and control hemispheres in terms of MC1 labelling but it appears that the MC1 labelling is considerably stronger and indeed rather different in the EC-Tau than in the EC-Tau/hAPP, especially in the dentate and EC which goes completely against the data reported in Fig 1. This is also largely the case in Fig. 7. Fig.8 legend doesn't seem to match the figure. Is this an

old version of the figure perhaps? It is very unclear what is being shown in B and D. Different coloured boxes mentioned in the legend don't seem to be a feature of this figure.

We thank the reviewer for their helpful criticisms regarding the tau immunostaining figures in our previously submitted manuscript. Former Figure 8B & D were indeed missing a y-axis and the legend was an outdated version for the figure. We are happy to report that we have revised and simplified our tau immunostaining results following chronic hM4Di DREADDs activation, and have included new data from additional EC-Tau/hAPP mice in updated Figure 5 (n=6 EC-Tau/hAPP mice). We have also removed EC-Tau mice from the analysis, eliminating data pooling in the immunohistochemistry quantification. Below are our comments on the specific critiques from Reviewer #3.

1.) The 16-month timepoint was chosen for our experiments on the basis that pathological tau (MC1+) had begun appearing in hippocampal subregions, suggesting propagation from a primary point of transgene expression (entorhinal cortex) into synaptically connected, downstream brain regions. However, we observed some variability in the levels of tau pathology in both EC-Tau and EC-Tau/hAPP mice at the 16-month timepoint, which is why we see varying levels of aggregated tau in mice from the same strain (e.g. EC-Tau/hAPP mice, former Figure 6F versus P, etc.). In the previous manuscript, we wanted to show as much of the histology as possible to illustrate the effects of DREADDs on tau, as the DREADDs-associated reduction in tau immunostaining was quite striking in some mice (former Fig 6F) but somewhat subtle in others (former Fig 6A).

We realize that focusing on DREADDs-mediated reductions in tau pathology resulted in a confusing presentation of the data where extreme cases of variability in tau pathology were shown. We do not feel this is representative of the effects we were trying to show. In general, we observed that EC-Tau/hAPP mice as a group had significantly increased tau pathology along the EC-HIPP network than age-matched, littermate EC-Tau mice. Data described in Figure 1 of our manuscript agrees with previously published data from our lab describing an effect of amyloid pathology on tau propagation in a similar mouse model (Khan *et al*., (2014) *Nature Neuroscience*. Figure 6C). Our data also agrees with data reported in a similar mouse line from Dr. Bradley Hyman's group (rTgTauEC x APP/PS1; Pooler *et al*., (2015) *Acta Neuropathologica Communications*. Figure 2). Figure 2 from Pooler *et al*., (2015) is shown below to illustrate the similarity in these results from different lab groups. Finally, we felt that within-subject comparisons of Aβ and tau immunoreactivity across hemispheres was the best way to test our hypotheses, which factor in inherent variability in pathology.

Pooler *et al* (2015) *Acta Neuropathologica Communications*

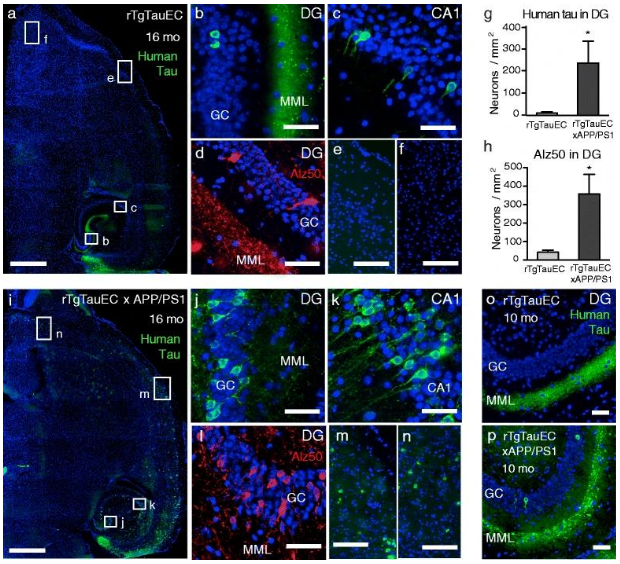

Figure 2. Propagation of tauopathy along neural circuits is exacerbated by amyloid pathology

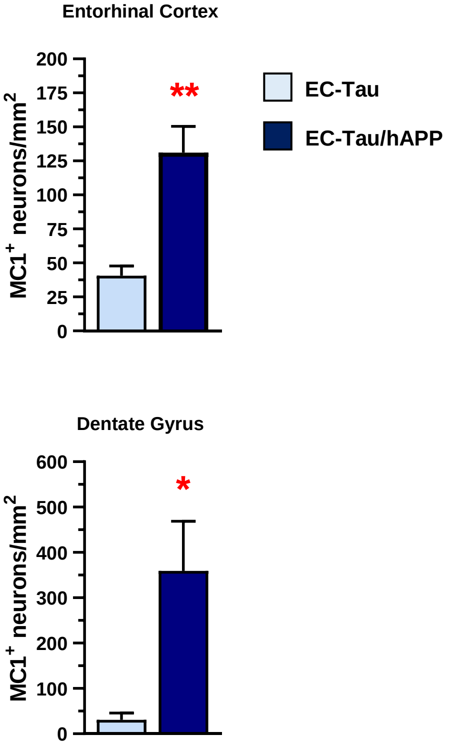

Discussion and Conclusions

Overall the paper largely supports previous data that suggests that the presence of raised amyloid beta or perhaps the presence of plaques increases Tau pathology and also increases activity of wide spiking neurones with the Tau pathology causing little, if any effect on activity. However unfortunately until the data are analysed per mouse rather than cells being pooled, very little else can be confidently concluded. Although the experiments are elegant, the sample sizes are too low considering the variability between animals for any confident conclusions in most cases. This is presumably due to the difficulty and cost of the experiments but unfortunately it undermines most of the conclusions. It is possible that the changes with DREADS are large enough and consistent enough to allow conclusions with these small sample sizes, if analysed per animal, but it is not possible to assess this from the data supplied. As many of the experiments (pathology, electrophysiology and behaviour) have been carried out on the same animals and as there is considerable variability between the animals in some cases, it may be that additional interesting information could be gleaned from correlating the outcomes of different experiments in each mouse. For example if the hypothesis that network activity increases the occurrence and spread of Tau pathology is correct, the 4 double transgenic mice should have very different pathology which should correlate with the very widely inter-mouse variability of activity of their wide spiking neurones.

If the sample sizes were increased and the data correctly analysed, without pseudoreplication, this could be a very interesting study. That Alzheimer's disease or other Tauopathies could be treated by just damping down network activity is however a slightly doubtful conclusion as the consequences on cognition of this treatment would presumably be rather undesirable.

We are happy to report that we have now increased our sample sizes for electrophysiological data (please see updated Figures 2, 3 and Supplemental Table 1) and analyze our data on a per mouse basis, discarding previous data pooling for these datasets. We are also happy to report that we now include immunostaining data from additional aged EC-Tau/hAPP mice that underwent chronic hM4Di EC DREADDs activation, increasing the sample sizes reported in the remaining Figures 4, 5 and Supplemental Figures 2, 3, 4 and 5.

Importantly, our study demonstrates that Aβ and tau pathology can be ameliorated to a degree by attenuating neuronal activity alone. We do not want to overreach with our conclusions, and thus do not conclude that AD can be treated by simply dampening neuronal activity. We state that attenuating activity can be a powerful tool if paired with other therapies that alleviate Aβ and tau aggregation (Page 12, Lines 452-454). Also, we state that additional studies will be required to determine if chemogenetic attenuation of neuronal activity in AD mouse models will have a positive effect on cognitive behavior (Page 12, Lines 463-465), which is an important step in validating an approach for treatment. While we do not report on cognitive behavior in this study, it is an area that we are currently investigating.

Reviewer #4, Hatim Zariwala:

The authors highlight a new transgenic line that shows pathological hallmark of AD – amyloid plaques and hyperphosphorylated tau in the anatomical regions associated with early AD. They dive further into exploring the phenomena of hyperexcitability in the entorhinal cortex and correlate that with the spread of tau. The in vivo electrophysiological work along with pathological analysis is a novelty of this paper. The study advances the functional effects of pathological hallmark observed in AD. I am in favor of publication of this paper after the authors have addressed the major concerns in this paper.

Major:

The introduction of Early and Late pathology in reference to DREADD stimulation is a bit confusing given low Ns in Fig 6 and later. Perhaps it should have been introduced in Fig 1, where the authors introduce the new transgenic line. Why this bimodal consequence of crossing the two lines? I would also like to see the MC1 staining data from Fig 1 presented on the scale of 0-2 for hemispheric distribution. AT8 and CP27 data would be a nice to have to build a complete picture for comparison later on.

We thank the reviewer for their helpful comments regarding the tau immunostaining figures in our previously submitted manuscript. Several of our reviewers felt that the presentation of Early and Advanced tau pathology in our immunostaining experiments was at best confusing to the reader and at worst possibly contradictory to our findings in Figure 1. Also, former Figure 8B & D were missing a y-axis and the figure legend was an outdated version for the figure. We apologize for this oversight. We are happy to report that we have revised and simplified our tau immunostaining results following chronic hM4Di DREADDs activation, and have included new data from additional EC-Tau/hAPP mice in updated Figure 5 (Total, n=6 EC-Tau/hAPP mice). We also show high resolution images at low (4X) and high (20X) magnification for two experimental EC-Tau/hAPP mice. We have also removed EC-Tau mice from the analysis, eliminating data pooling in the immunohistochemistry quantification.

In general, we observed that 16-month EC-Tau/hAPP mice as a group had significantly increased tau pathology along the EC-HIPP network than age-matched, EC-Tau littermates. Below, we show MC1+ immunoreactivity data presented in Figure 1, with right versus left hemisphere differences in MC1+ neurons/mm^2 represented as a ratio and plotted on a 0-2 scale (left panel). This data shows that on average, MC1 cell counts in DG do not significantly differ across hemispheres. However, chronic activation of hM4Di EC DREADDs resulted in a decreased Right / Left Hemisphere ratio for our MC1+ threshold analysis (Figure 5O and below right), which detects MC1+ immunoreactivity (see Supplemental Figure 4D).

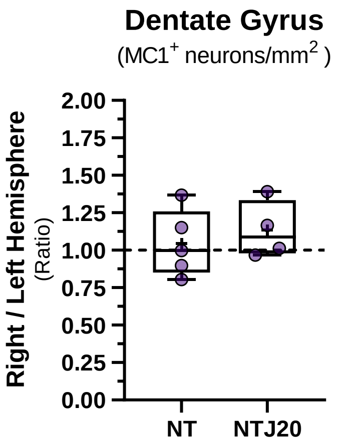

16-month EC-Tau
One-sample *t* test: $t_{(4)}$=0.440, p = 0.6824

16-month EC-Tau/hAPP
One-sample *t* test: $t_{(3)}$=1.404, p = 0.2551

Figure 5O, left panel

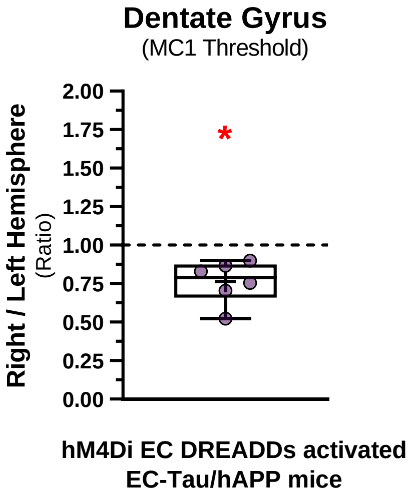

In the previous version of our manuscript, we wanted to show as much of the tau histology as possible to illustrate the effects of DREADDs on tau, as the DREADDs-associated reduction in tau immunostaining was quite striking in some mice (former Fig 6F) but somewhat subtle in others (former Fig 6A). We realize that focusing on DREADDs-mediated reductions in tau pathology resulted in a confusing presentation of the data where extreme cases of variability in tau pathology were shown (Early and Advanced). We do not feel this is representative of the effects we were trying to show. We now feel that the images and immunostaining data shown in Figure 5 of our revised manuscript better represent the effects we observed after chronic DREADDs activation.

I think if the hemispheric distribution is plotted to reflect the effect of stimulation with DREADD than the Ns has to N = 2 instead of N = 4 (as N = 4 is mentioned in the caption). How is N = 2 distributed in the Panel B and D of Fig 8 (which circles are coming from the same animal). In general, the data is limited and the variability (intra-subject, intra-section and intra-hemisphere) cannot be discerned from the way the data is presented. Ideally more Ns are needed to stake a claim. Unless data from subjects in Fig 1 could help to compare with Fig 8. This will be a major take away from the paper for those who want to test pharmacological effect on hyperexcitability. It is important that the authors provide a good benchmark data for future studies. I am accepting of the fact that DREADD is not a perfect tool so things may not look very compelling, but my concerns are more related to the transgenic line and limited data in fig 6 onwards to drive the interpretation.

Minor:

1. The introduction should have a few sentences on the clinical perspective. There have been several failed clinical studies that lower amyloid levels yet no positive outcome in patients. To advance this research the authors should discuss the chronology of events in their mouse model amyloid deposition > hyperactivity > tau progression as it might relate to the clinical pathology findings. Another item on my wish list is to assess any cognitive consequences of hyperexcitability in these mice? Since there are no good biomarkers to assess hyperexcitability, perhaps cognitive effects can be readily assessed in patients. Perhaps the authors can speak to that in driving this research forward.

We agree with the reviewer on the importance of examining our results from a clinical perspective. However, we prefer to address these points in the Discussion (Page 12, Lines 452-469), where we can explore them in the context of our experimental results. Specifically, we acknowledge that current clinical trials aimed at eliminating/reducing Aβ have failed to meet cognitive endpoints. We speculate that this may be due to ineffective amelioration or exacerbation of neuronal network disturbances, which we feel is an important feature of Alzheimer's disease. Our pre-clinical data in mice demonstrates the importance of examining network disturbances in addition to Aβ and tau pathology, and offer new data demonstrating that reducing neuronal activity can achieve a degree of pathology amelioration along an important neuronal circuit.

We are very interested in reporting on cognitive behavioral consequences of neuronal network dysfunction in the EC-Tau/hAPP mouse line. However, experiments designed to assess cognitive behavior will be performed in additional studies to address this point. Our current, revised manuscript focuses on electrophysiological and behavioral characterization of the EC-Tau/hAPP mouse line, and utilizes a chemogenetic approach to alleviate some AD pathology.

Interestingly, a number of "digital biomarkers" are being explored as useful diagnostic tools for network dysfunction associated with MCI or prodromal Alzheimer's disease. These include actigraphy and traditional polysomnography, which can identify network dysfunction related to sleep disturbances, and EEG which can capture epileptiform-like activity. Our results stress the importance of assessing network activity in pre-clinical and clinical studies of AD, in addition to measuring impact on pathology.

Information on how the transgenic mice were generated (Hets X Hets or HOM s X Homs) and how the double

transgenic were selected is completely missing. Some text should be added along with ease (or lack of ease) of breeding.

We now provide additional information regarding generation of the triple transgenic EC-Tau/hAPP mouse line and breeding scheme in the methodology. Please see Page 14, starting at line 486.

3. Page 6: Could the loss of interneuron hyperactivity be the explanation of the increased network hyperactivity seen in ECTau/APP mice compared to APP mice only? Related to that question, is there a loss of interneuron in EC in the EC-Tau/APP mice?

We are intrigued by the possibility that impaired EC interneuronal firing could be responsible for overall hyperactivity in the region. A loss of inhibitory signaling onto excitatory neurons in EC-Tau/hAPP mice could translate to unregulated excitation and production/release of Aβ and tau. However, additional experiments that specifically target EC inhibitory interneurons will need to be performed in order to address this mechanistic question. What we know now from our immunostaining data is that interneuron populations appear spared from accumulating tau pathology when compared to excitatory cells (Fu *et al.*, (2017) *Neuron*; Fu *et al.*, (2019) *Nature Neuroscience*), though this does not necessarily mean that normal interneuron firing capabilities are also spared.

4. Supplemental Fig 2: Since this was in n = 1 mouse per group, could the authors provide the n of cells and sense of distribution of the spike count per cell recorded.

We now provide additional time-course data (n=6 mice) for acute DREADDs activation in updated Supplemental Figure 3A in our revised manuscript. Below is the time-course data shown in Panel A, along with the statistical testing report.

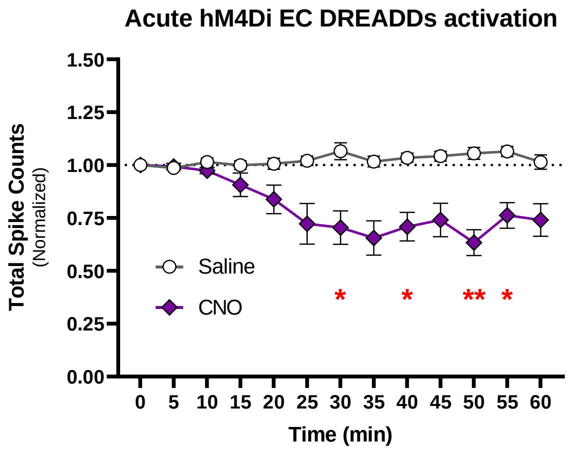

Two-way ANOVA w/ repeated measures

Interaction $F_{(11,110)} = 5.448$, $p < 0.001$
Time $F_{(2.4,23.5)} = 3.112$, $p > 0.05$
Drug $F_{(1,10)} = 30.490$, $p < 0.001$

Sidak's multiple comparisons test

CNO vs Control, 30 min; $p < 0.05$
CNO vs Control, 40 min; $p < 0.05$
CNO vs Control, 50 min; $p < 0.01$
CNO vs Control, 45 min; $p < 0.05$

In order to get a sense of the population activity changes that follow DREADDs activation, we feel it is best to evaluate total spike counts across tetrodes for each mouse. However, we would like to provide an example of a single-unit recorded from an EC-Tau mouse injected with 5MG/KG CNO. One can see that the activity changes closely match that of the population change for this mouse and the AVG FR decreases after CNO injection.

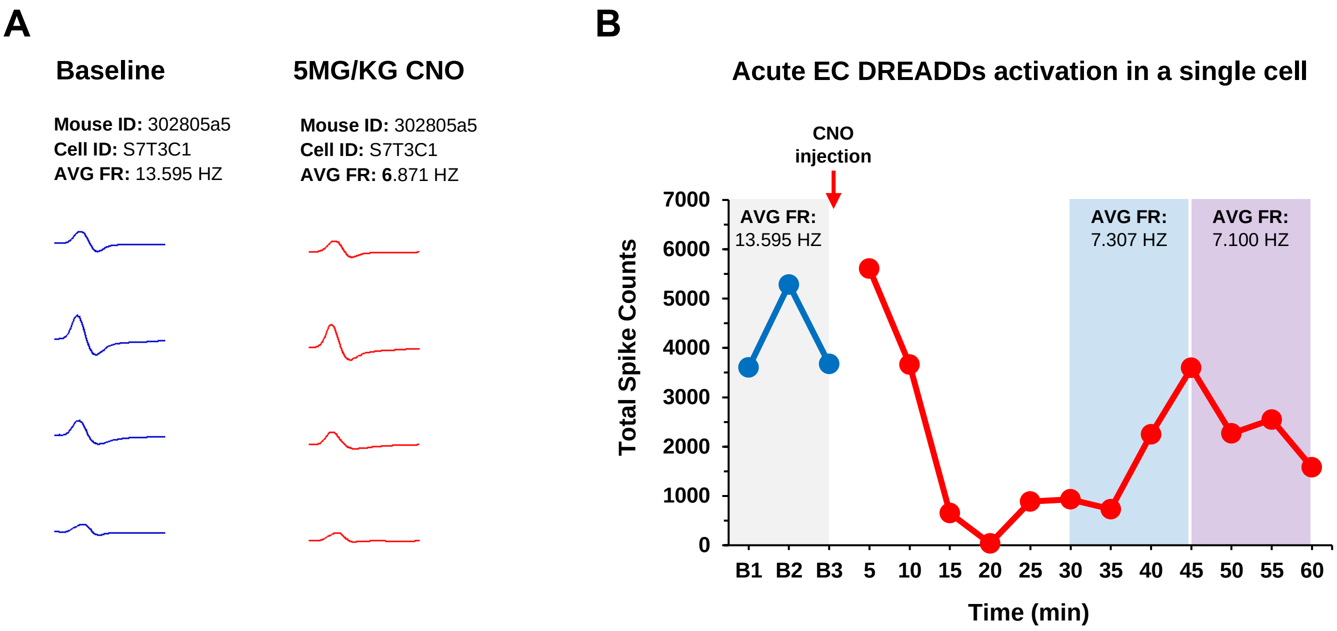

Acute hM4Di DREADDs activation reduces neuronal activity in an EC single-unit. Example of the typical response from an EC neuron after injection of CNO. Mouse ID: 302805a5, 16-month old EC-Tau. ***A***. The waveform of one EC single-unit is shown at Baseline (blue) and after 5 mg/kg CNO injection (red). Mouse ID, Cell ID and the AVG FRs per condition are shown on top of waveforms. ***B***. The total spike counts for EC single-unit (S7T3C1) are shown pre- and post-CNO injection. Baseline spike counts are shown in Blue. Post-CNO spike counts are shown in Red. AVG FRs are shown for 15 min epochs, depicted by shaded regions. AVG FR for this cell is reduced by ~ 50% after CNO injection compared to Baseline.

Updated Supplemental Figure 3D also features data from additional animals (n=8 mice) and is shown below along with the statistical testing report.

Repeated measures one-way ANOVA
$F_{(2,7)} = 7.079$, $p < 0.05$

Dunnett's multiple comparisons test
45-60 min vs Baseline, $p < 0.05$

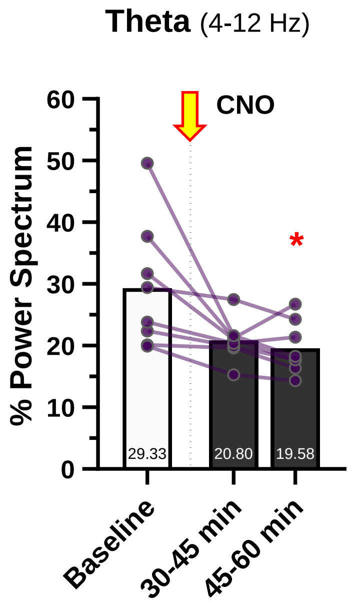

5. Line 613: some text is missing to explain preparation of CNO. Also, FYI this is what I am trying to put together for side-to-side hemispheric tau pathology

We thank the reviewer for pointing out this oversight. The sentence has now been revised to read, "Clozapine-n-oxide (CNO; Sigma Aldrich, USA) was dissolved in sterile saline with 0.05% DMSO."
