# Supplementary material for: Chemogenetic attenuation of neuronal activity in the entorhinal cortex reduces Aβ and tau pathology in the hippocampus
Source: PLoS Biol. 2020 Aug 21;18(8):e3000851. doi: 10.1371/journal.pbio.3000851 (PMC7467290; doi:10.1371/journal.pbio.3000851)
Supplement: Attachment — Submitted filename: Rodriguez et al - PLOS Bio_3 - Responses.pdf [file pbio.3000851.s009.pdf]

POINT BY POINT RESPONSES

Reviewer #5 (new reviewer): In this manuscript, authors Rodriguez et al. investigate pathology and network activity in the entorhinal cortex (EC) and downstream hippocampal (HP) pathway in a novel transgenic model overexpressing mutant human APP and EC-specific Tau. Their results suggest that amyloid drives hyperexcitability and the propagation of tau pathology to the HP. Following this logic, the author's chemogenetically decreased neuronal activity in the EC and showed that that this manipulation reduced hyperphosphorylated and abnormally conformed tau in some downstream regions. This work provides interesting data on the interplay between Aβ and tau pathology in Alzheimer's disease. Overall the paper is quite good, and the authors are to be commended for the new data and extensive revisions they added in response to the first set of reviews. Addressing a few additional points could further improve the impact of this work.

1. Figure 1 shows amyloid and tau pathology at multiple time points in the EC-Tau/hAPP cross, as well as in the EC-Tau mice at 16 months. It would be helpful to also show results from the hAPP transgenic mice.

> We unfortunately do not have horizontal brain sections from 23-month hAPP/J20 mice to generate a complimentary set of 6E10 immunostaining panels like those in Figure 1A. However, we did examine hippocampal 6E10+ immunoreactivity in horizontal brain sections from 16-month EC-Tau/hAPP mice (n=9) and age-matched hAPP mice (n=6) to determine the effect of human mutant tau expression on hAPP/Aβ deposition. For this data and an explanation of the results, please see our response to Reviewer #6, who specifically asked for the group comparison.

2. The box legend in Figure 2 illustrating the genotypes is difficult to locate, and should be repositioned closer to 2B.

> We thank Reviewer #5 for bringing this to our attention. This was a mistake in the uploaded version of Figure 2 and has now been corrected.

3. The CP27 staining in Figure 5A-E suggests that the tau pathology that "spread" from the EC to the hippocampus was composed of mutant human tau. Can the authors comment on whether corrupted endogenous mouse tau was also recruited?

> Murine tau can form filamentous aggregates in the brains of rTg4510 mice overexpressing $hTau^{P301L}$ in forebrain, though it would appear that recruitment of corrupted mouse tau occurs later in the lifespan relative to MC1+ aggregates (Ren *et al*., 2014. *Journal of Alzheimer's disease*). It has also been shown that purified tau fibrils from AD brains can trigger tau deposition in young wild-type mice (C57BL6 and C57BL6/C3H F1) along anatomically connected brain regions without neuron loss (Guo *et al*., 2016. *Journal of Experimental Medicine*).
>
> Based on these reports and others, we speculate that corrupted murine tau in the entorhinal cortex of EC-Tau/hAPP mice can be recruited in the propagation of tau pathology, but the extent and severity of murine tau participation in impaired neuronal activity is unknown. Additional immunostaining experiments using mouse tau-specific antibodies would be required to address this question.
>
> *Guo et al. "Unique pathological tau conformers from Alzheimer's brains transmit tau pathology in nontransgenic mice". Journal of Experimental Medicine. 213(12). (2016): 2635-2654.*
>
> *Ren et al. "Endogenous tau aggregates in oligodendrocytes of rTg4510 mice induced by human P301L tau". Journal of Alzheimer's Disease. 38.3 (2014): 589-600.*

4. In line 220, it is unclear what "Single, high dose CNO (5 and 10mg/kg, i.p.) injections reliably induced DREADDs expression" means. The expression of DREADDs is independent of ligand administration. In the response to reviewers, it was stated that this sentence had been changed, but it still reads the same in the revised version I received.

This was an unfortunate oversight but has now been corrected in this revised manuscript. The sentence now reads, "Single, high dose CNO (5 and 10mg/kg, i.p.) injections reliably induced DREADDs activation and altered EC neuronal activity (Supplemental Figure 3A-B) and % theta power (Supplemental Figure 3C-D), providing valuable metrics for detecting chronic DREADDs activation in vivo" (Lines 314-16 in the clean version of the manuscript).

5. Figure 4 illustrates a reduction in spiking. It is not clear whether this reduction was seen in NS (inhibitory) or WS (excitatory) neurons, or both. It would be helpful to show data separated by neuron type. Finally, was the reduction in 6E10+ pathology also seen in the EC, or just the hippocampus?

We chose to represent the chronic effects of hM4Di EC DREADDs activation by tracking the total spike counts in our neuronal recordings over time (% of Baseline). This was preferred over calculating an average firing rate value per mouse, as changes in spike counts are less prone to variability caused by gross changes in the number of neurons detected over time (CNO sessions versus Baseline) or in any unbalanced number of neuronal subtypes (NS or WS) detected. In our previous Figure 4A, we showed that chronic hM4Di EC DREADDs activation reduced the total spike counts in our recordings (Total, gray bars) and from separated neuronal sources (Separated, blue circles) over time. These changes in spike counts are reflective of changes to EC population activity after CNO treatment and not to individual neuron subtypes.

Nonetheless, we agree with Reviewer #5 and #6 that it would be useful to show the effects of chronic CNO-treatment on individual neuron subtypes in additional to population spiking activity and network activity (% Theta Power). We now present a revised main Figure 4 in the manuscript and have updated the Results, Discussion and Methods sections accordingly. Revised Figure 4 appears below.

We found that chronic hM4Di EC DREADDs activation reduced % Theta power in EC-Tau/hAPP mice (n=8 total: n=4 female, n=4 male) at Week 6 of CNO-treatment vs Baseline (Figure 4A). Repeated measures ANOVA: $F_{(7,21)} = 4.697$, $p < 0.05$. Dunnett's multiple comparisons test: Week 4 vs Baseline, $p > 0.05$; Week 6 vs Baseline, $p > 0.05$; Week 6 vs Baseline, $p < 0.05$. Chronic EC hM4Di DREADDs activation also reduced total spiking activity in EC-Tau/hAPP mice (Figure 4B). Repeated measures ANOVA: $F_{(7,21)} = 11.270$, $p < 0.001$. Dunnett's multiple comparisons test: Week 4 vs Baseline, $p > 0.05$; Week 6 vs Baseline, $p < 0.05$; Week 6 vs Baseline, $p < 0.001$. Automated spike sorting and manual cluster cutting was then performed on neuronal spiking data from two recording sessions per EC-Tau/hAPP mouse (Baseline and Week 6, CNO). Chronic EC hM4Di DREADDs activation reduced Total EC Neuron average firing rates at Week 6 vs Baseline (Figure 4C). Paired t-test: $t_{(6)} = 4.976$, $p = 0.0025$. Single-units were then categorized as Narrow-Spiking or Wide-Spiking according to spike width (Figure 4D-E). Average firing rates were reduced at Week 6 vs Baseline in both Narrow-Spiking EC neurons (Paired t-test: $t_{(6)} = 2.635$, $p = 0.0388$) and Wide-Spiking EC neurons (Paired t-test: $t_{(6)} = 2.804$, $p = 0.0310$).

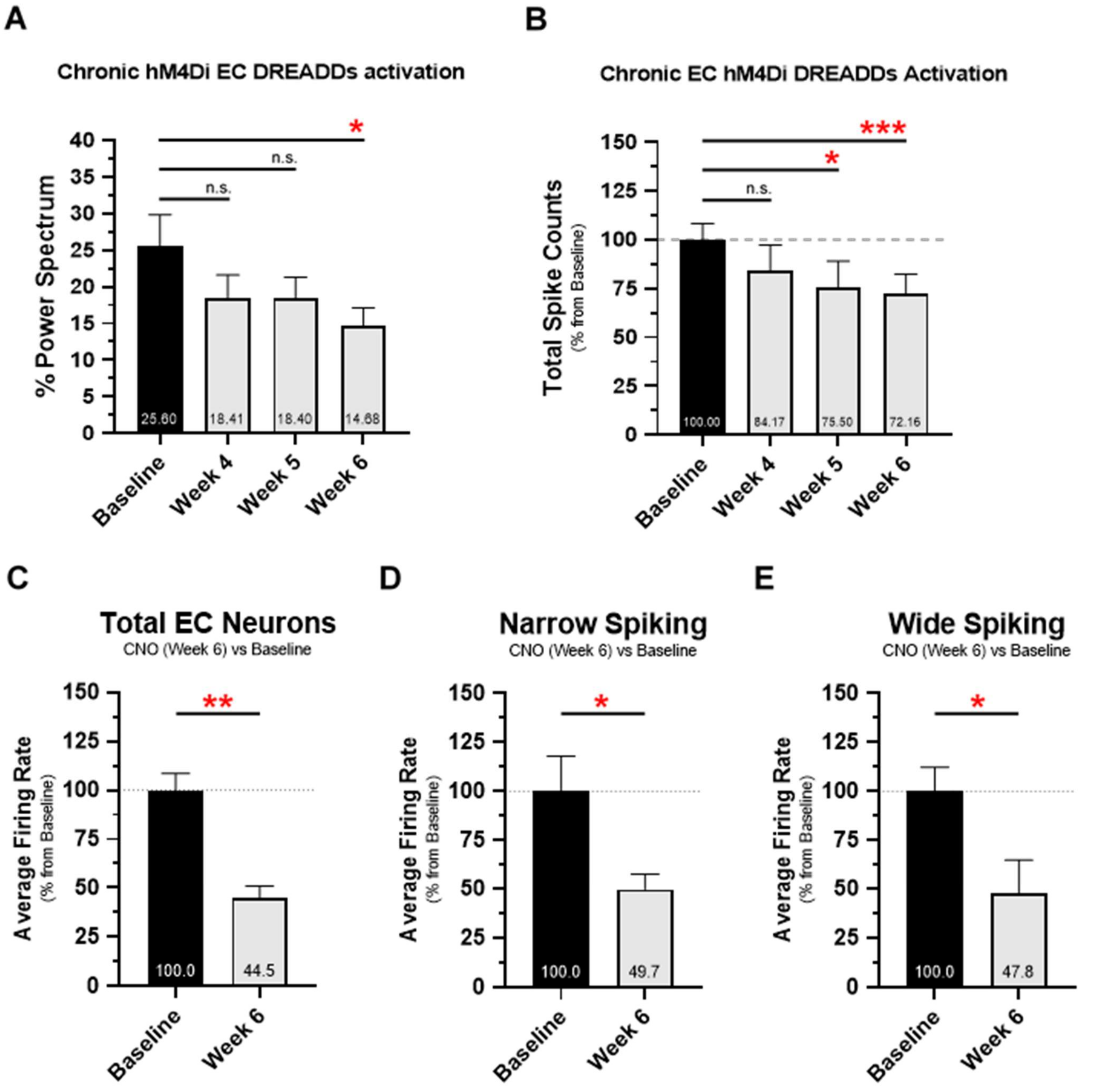

Figure 4. Chronic hM4Di EC DREADDs activation reduces EC neuronal network activity and single-unit firing rates at 6 weeks. 16-month EC-Tau/hAPP mice were subjected to 6-weeks of DREADDs activation via osmotic minipump (CNO, $1mg^{-1}/kg^{-1}/day^{-1}$). *In vivo* neuronal activity was examined at 4-, 5- and 6-weeks of CNO treatment and compared to baseline activity (pre-surgery) measures). ***A.*** Chronic EC hM4Di DREADDs activation reduced % Theta power in EC-Tau/hAPP mice (n=8 total: n=4 female, n=4 male). Repeated measures ANOVA: $F_{(7,21)} = 4.697$, $p < 0.05$. Dunnett's multiple comparisons test: Week 4 vs Baseline, $p > 0.05$; Week 5 vs Baseline, $p > 0.05$; Week 6 vs Baseline, $p < 0.05$. ***B.*** Chronic EC hM4Di DREADDs activation also reduced total spiking activity in EC-Tau/hAPP mice. Repeated measures ANOVA: $F_{(7,21)} = 11.270$, $p < 0.001$. Dunnett's multiple comparisons test: Week 4 vs Baseline, $p > 0.05$; Week 5 vs Baseline, $p < 0.05$; Week 6 vs Baseline, $p < 0.001$. ***C.*** Automated spike sorting and manual cluster cutting was performed on neuronal spiking data from two recording sessions per EC-Tau/hAPP mouse (Baseline and Week 6, CNO). Chronic hM4Di EC DREADDs activation reduced Total EC Neuron average firing rates at Week 6 vs Baseline. Paired *t*-test: $t\ (6) = 4.972$, $p = 0.0025$. ***D-E.*** Single-units were then categorized as Narrow-Spiking or Wide-Spiking according to spike width. Average firing rates were reduced at Week 6 vs Baseline in both Narrow-Spiking EC neurons (Paired *t*-test: $t\ (6) = 2.635$, $p = 0.0388$) and Wide-Spiking EC neurons (Paired *t*-test: $t\ (6) = 2.804$, $p = 0.0310$). Graphical representations appear as mean ± SEM. Normalized data is shown as a % of Baseline. * $p < 0.05$, ** $p < 0.01$. *** $p < 0.001$.

As to the reviewer's last point, we found that threshold analysis for 6E10 in the entorhinal cortex was complicated by the damage to tissue sections caused by the tetrodes. This can be seen in Supplemental Figure 4A, which we have reproduced for the Reviewer below (Rebuttal Figure 1, *middle and bottom panels*) along with an additional section from this mouse (*top panels*). Notably, the intense dark brown staining around the tetrode tracts did not allow clean right versus left hemisphere comparisons in the entorhinal cortex. This complication was present in tissue sections from our other EC-Tau/hAPP mice as well.

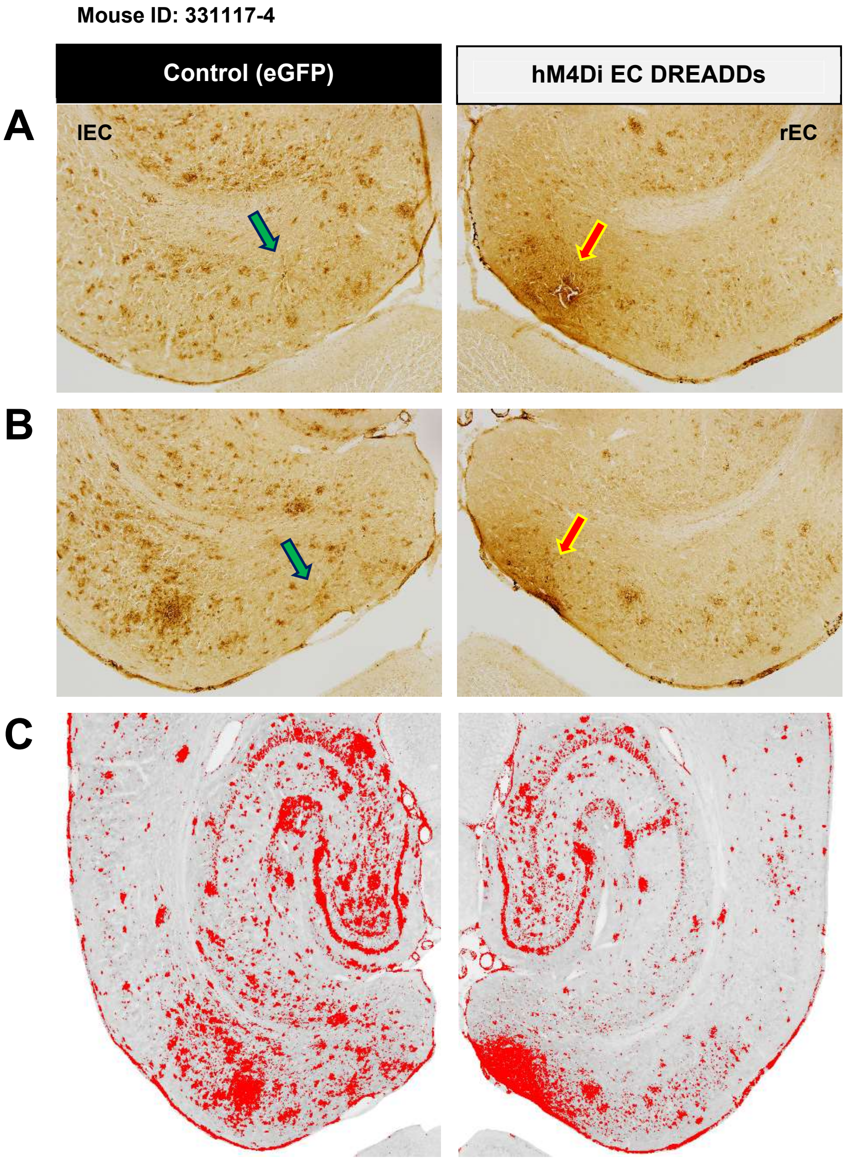

Rebuttal Figure 1. Complications to 6E10+ immunoreactivity analysis in the EC. Two horizontal brain sections from Mouse ID: 331117-4 are shown after 6E10+ immunostaining. In the right hemisphere, red arrows indicate the location of the tetrode tracts. In the left hemisphere, green arrows indicate the location of the viral (control eGFP) injection needle tract. ***A.*** Intense DAB reactivity can be seen around the site of the tetrode tract. ***B.*** High magnification images from sections shown in Supplemental Figure 4A (***C***), where dark staining is evident at the terminal end of the tetrode tract. This intense DAB reactivity was prohibitive to our image analysis of Aβ coverage in the EC.

6. The authors should be conservative about generalizing their data, since they only examined particular mutations and one brain region, and there are different ways of measuring neuronal activity. There are reports that tau can cause hyperexcitability, even though it was not evident in this study. For example, how do the authors think about their data in the context of Sohn et al., Neuron 104:458-70? How do the authors think about their data given that tau pathology in human Alzheimer's disease is composed of wild-type tau, not P301L mutant tau? Some discussion of these issues should be added to the manuscript.

We agree with the reviewer that we do not wish to overgeneralize our findings, especially when reporting tau-associated EC activity levels. Importantly, we include a statement cautioning our readers to interpret EC-Tau electrophysiology data carefully (Lines 584-87, clean manuscript version). We have carefully chosen to discuss the results as it pertains to Aβ and tau pathology along the EC-HIPP circuit in our mouse model, and now include more detailed discussion of neuronal dysfunction (e.g. hypoactivity and hyperactivity) related to tau in the remainder of the paragraph. We also include several new citations where the reader is referred for data describing tau-associated hyperexcitability in various mouse models.

Our data in the context of results described in Sohn *et al* (2019) *Neuron*

Sohn *et al* (2019) describe a potential mechanism for tau-mediated hyperexcitability in human iPSC-derived neurons, wherein the frontotemporal dementia (FTD)-associated $hTau^{V337M}$ mutation impairs axon initial segment (AIS) plasticity and homoeostatic excitability. We are intrigued by the possibility that $hTau^{P301L}$ also impacts the AIS, which is a specialized cellular compartment responsible in part for regulating the cell's membrane potential and action potential initiation. However, we do not know the state of the AIS in EC-Tau/hAPP mice and thus cannot comment on whether the effect of $hTau^{P301L}$ on the AIS differs versus Tau^{V337M}. Jorge Palop's work demonstrating reduced Nav1.1 functionality in hAPP/J20 mice offers some mechanistic clue as to the root of neuronal hyperactivity in our EC-Tau/hAPP model, though the joint overexpression of $hTau^{P301L}$ certainly complicates a mechanistic explanation of the data. Nav1.1 is a voltage-gated sodium channel subunit that is predominantly expressed in interneurons at the AIS, and its depletion is associated with aberrant network activity. We are therefore careful not to speculate too much about the mechanisms underlying single-unit or network dysfunction in our mice, as additional experiments are required to define exactly how Aβ and tau interact at the cellular level to impair neuronal activity. Another important consideration when comparing our *in vivo* results to the *in vitro* results of Sohn *et al* (2019) is the composition of excitatory versus inhibitory neurons in the model. We report approximately 60% excitatory neurons and 40% inhibitory neurons in our EC recordings. In contrast, Sohn *et al* (2019) utilized a protocol to convert human iPSCs into homogenous excitatory cortical neurons with minimal variability.

We realize that no *MAPT* mutations have been identified in patients with autosomal dominantly inherited AD. However, we think that the P301L human mutant tau targeted preferentially in the EC neurons offers important insight into region specific tau pathology which currently is not possible with other mouse models. Future studies will have to focus on understanding the role of different tau forms in driving EC pathology.

Reviewer #6 (new reviewer, also as an attachment): In this manuscript, Rodriguez et al. use a new transgenic mouse line expressing both Tau and hAPP in the entorhinal cortex (EC) to show that hAPP aggravates tau aggregation in the entorhinal cortex and accelerates pathological tau spread into the hippocampus. The authors identify hAPP/Aβ, and not tau, as the major trigger for neuronal hyperactivity and impaired theta in the EC. Finally, the authors use a chemogenetic approach to attenuate neuronal hyperactivity in the EC and they provide evidence for reduced hAPP/Aβ accumulation and reduced pathological tau spread downstream in the hippocampus. Overall this study nicely complements previous studies showing abnormal activity patterns in the context of AD, and its link with disease progression.

It is a revision of a manuscript for which the main criticisms dealt with small sample size and with quantification methods. The authors have made the appropriate efforts to answer to most of the concerns of the reviewers. They have increased the sample size when necessary, and improved the presentation. The manuscript should now be potentially suitable for publication, provided minor revisions indicated below:

Figure 1A - It is quite unfortunate that there is no quantification of the plaques or hAPP/Aβ immunoreactivity, in the different areas and models as there is for MC1 immunoreactivity. See line 241, this has been done in part of the CNO experiments.

We examined hippocampal 6E10+ immunoreactivity in sections from 16-month EC-Tau/hAPP mice (n=9) and age-matched hAPP mice (n=6) to determine the effect of human mutant tau expression on hAPP/Aβ deposition. As noted by Reviewer #6, sections from three hAPP mice were already included in our analysis of Aβ Coverage in Control Conditions (Figure 4F). Data from these three mice were combined with data from three independent 16-month hAPP mice included in the original version of our manuscript. This data was excluded from our main Figure 4 in the second version of our manuscript based on reviewer recommendations not to mix genotypes. Importantly, only 6E10+ immunoreactivity in the left hippocampus were used for analysis. We explain these reasons in the caveat section at the end of this response.

An unpaired t-test found increased Aβ coverage (%) in hippocampus of 16-month EC-Tau/hAPP mice versus age-matched hAPP mice ($p < 0.01$) (Rebuttal Figure 2). This data agrees with anti-Aβ immunostaining (AW7 antibody) in a similar mouse model, 16-month old rTgTauEC x APP/PS1 mice, described in Pooler *et al* (2015) *Act Neuropathologica Communications*. Figure 5 data from this paper is shown below our Rebuttal Figure 2 data. Briefly, increased Aβ plaque size and plaque burden was seen in somatosensory cortex and entorhinal cortex of 16-month rTgTauEC x APP/PS1 mice (n=11) versus age-matched APP/PS1 mice (n=4).

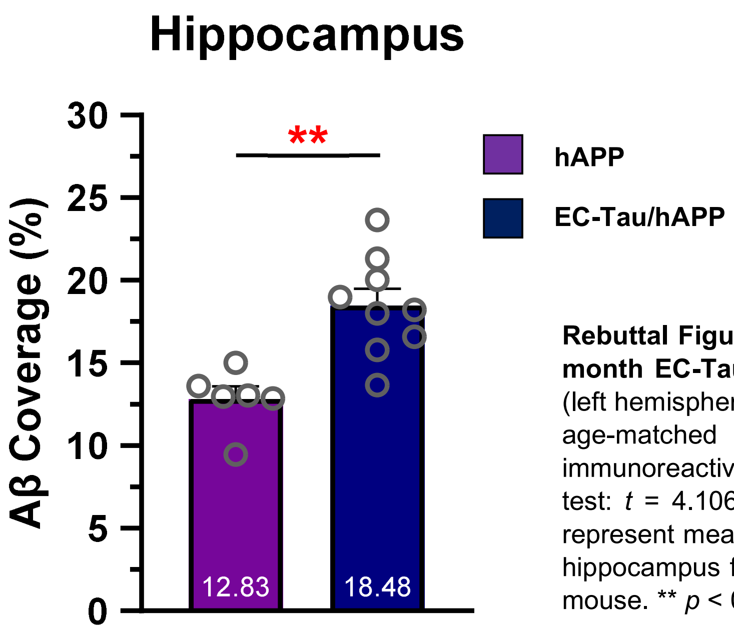

Rebuttal Figure 2. Hippocampal Aβ coverage is increased in 16-month EC-Tau/hAPP mice. We compared 6E10+ immunoreactivity (left hemispheres) in the hippocampus of EC-Tau/hAPP mice (n=9) to age-matched hAPP mice (n=6). We found elevated 6E10+ immunoreactivity in hippocampus of EC-Tau/hAPP mice. Unpaired t-test: $t = 4.106$, $p = 0.0012$. Graphs and numerical values in figure represent mean ± SEM for the averaged % 6E10 immunoreactivity in hippocampus from three independently processed brain sections per mouse. ** $p < 0.01$.

Pooler *et al* (2015) *Acta Neuropathologica Communications*

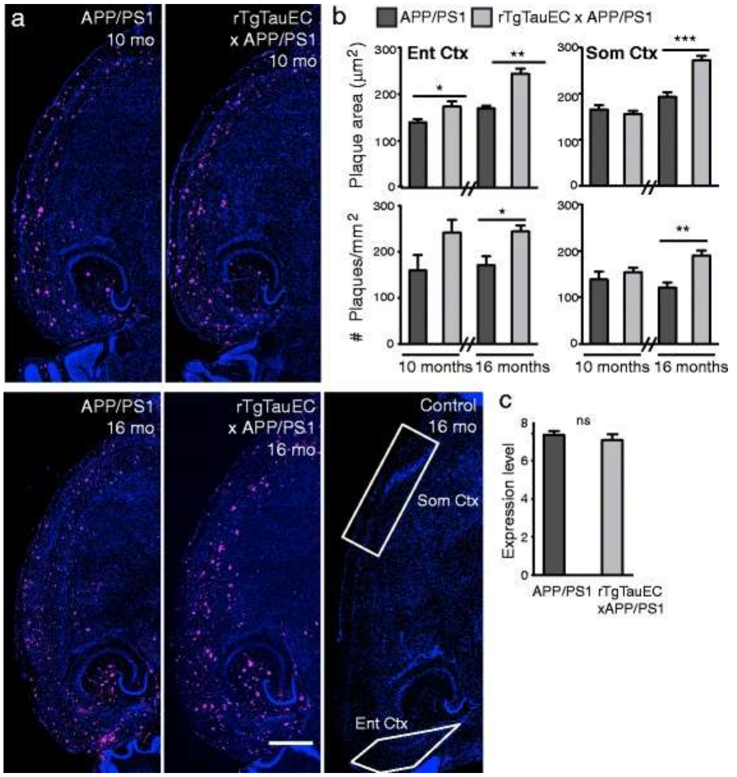

Figure 5: Expression of human tau increases amyloid burden

Caveats to the data: Our comparison of hippocampal 6E10+ immunoreactivity between sections from EC-Tau/hAPP mice and hAPP mice was restricted to data from the left brain hemispheres only. As evident from the data table provided below, all fifteen mice expressed hM4Di DREADDs in the right EC and all but three mice in the analysis underwent chronic hM4Di EC DREADDs activation with CNO-treatment (1 mg/kg/day) via osmotic minipump. Only the three 16-month hAPP mice noted by Reviewer #6 were left untreated.

We hesitate to include this 6E10+ immunostaining analysis in our manuscript because of the potential for confounding factors, which include the following: 1) off-target effects of chronic CNO treatment on Aβ pathology in the left hemisphere of n=12 of these mice, as CNO is delivered systemically, 2) the large imbalance in female mice between groups, and 3) potential of attenuated neuronal activity in right EC impacting left hippocampal Aβ pathology via cross-hemispheric connectivity. We acknowledge that the probability of these factors impacting our data may be minimal. Nonetheless, it is important to recognize these caveats. We will include the data in the manuscript as supplemental data upon recommendation by the journal editors and/or reviewers.

Data Table 1. Treatment conditions per mouse in Rebuttal Figure 2

Mouse ID	Genotype	Hemisphere	DREADDs Expression	CNO dose	
262031a3	hAPP	Left	Yes (right EC)	no CNO	Male
271031-2	hAPP	Left	Yes (right EC)	no CNO	Female
301031a2	hAPP	Left	Yes (right EC)	no CNO	Male
871031a2	hAPP	Left	Yes (right EC)	1 mg/kg/day; pump	Male
871031a3	hAPP	Left	Yes (right EC)	1 mg/kg/day; pump	Male
871031-5	hAPP	Left	Yes (right EC)	1 mg/kg/day; pump	Female
331117-1	EC-Tau/hAPP	Left	Yes (right EC)	1 mg/kg/day; pump	Female
331117-4	EC-Tau/hAPP	Left	Yes (right EC)	1 mg/kg/day; pump	Female
351117-3	EC-Tau/hAPP	Left	Yes (right EC)	1 mg/kg/day; pump	Female
311117a2	EC-Tau/hAPP	Left	Yes (right EC)	1 mg/kg/day; pump	Male
31117-1	EC-Tau/hAPP	Left	Yes (right EC)	1 mg/kg/day; pump	Female
101117-2	EC-Tau/hAPP	Left	Yes (right EC)	1 mg/kg/day; pump	Female
101117-4	EC-Tau/hAPP	Left	Yes (right EC)	1 mg/kg/day; pump	Female
51117a3	EC-Tau/hAPP	Left	Yes (right EC)	1 mg/kg/day; pump	Male
111117a1	EC-Tau/hAPP	Left	Yes (right EC)	1 mg/kg/day; pump	Male

Figure 1D - What is the use of these panels, and what differences with middle panels in 1A? In addition, what is the meaning and utility of the thick black lines?

The images in Figure 1D show representative 6E10+ immunostaining in horizontal brain sections from two 16-month EC-Tau/hAPP mice (Mouse IDs: 250704-1, 250704-2). When compared to the images in Figure 1C, the images provide our readers with a clear distinction in accumulated Aβ pathology in the EC-HIPP regions between the two genotypes (EC-Tau/hAPP versus EC-Tau). The 6E10+ images, along with that from Mouse ID 271117a2 in Figure 1A, provide our readers with representative Aβ pathology in three independent 16-month EC-Tau/hAPP mice. Finally, they also compliment MC1+ immunostaining images from the same set of mice in panels 1E & 1F.

The dark black lines over the two images simply served to indicate that the two mice are from the same genotype group. However, as this was confusing to the Reviewer and potentially to readers, we have modified the lines to sit over individual sections in this revised manuscript.

Figure 4 and 5. The effect of chemogenetic attenuation of EC neuronal activity on Aβ and Tau pathology is perhaps the most original result of the paper. The effect occurs on 16 months-old EC-Tau/hAPP mice which already exhibit significant level of pathology. A treatment with CNO for 6 weeks diminishes the level of both amyloid (Fig4D, E) and Tau (Fig5J, O). Thus, CNO does not inhibit the appearance of AD-related hallmarks but rather seems to partially revert the pathology. Surprisingly, there is no mention about the kinetic of the events and about the possible mechanisms involved: inhibition of Ab production and Tau phosphorylation, versus clearance of plaques and tangles. At minimum, this should be addressed in the discussion.

We chose to focus on conclusions drawn from the data in our manuscript. We found reductions in EC theta modulation and total spiking activity after chronic DREADDs activation (Figure 4A-B), in addition to reduced 6E10+ and tau immunoreactivity in the ipsilateral hippocampus (Figures 5-6). Thus, we hypothesized that these reductions in pathology were due to decreased production and synaptic release of Aβ and tau, which is tightly linked to neuronal activity. We were originally hesitant to include in our manuscript speculation into underlying mechanisms of Aβ and tau clearance, as we have not performed those experiments and cannot state with confidence what events occurred to facilitate clearance. Nonetheless, we agree with the reviewer that some speculation is warranted above the suppression of activity dependent Aβ and tau production/release.

We now discuss three possible underlying mechanisms of DREADDs-mediated Aβ and tau reduction in a new paragraph in the Discussion (Lines 634-58, clean manuscript version). These three mechanisms include 1) enhanced microglial reactivity at Aβ plaques, 2) increased activity of traditional degradation pathways and 3) increased clearance of Aβ and tau via the glymphatic system. All three points provide interesting avenues of research into Aβ and tau interactions as they relate to neuronal activity in the brain, and we are careful to inform our readers that additional studies are warranted to define the mechanisms of reduced pathology.

Line 226 - The authors should provide a comparison between the levels of neuronal activity (and theta power) attained after CNO treatment, and those observed in WT mice at the same age. Does the CNO treatment lead to normalization of neuronal activity? Or hypoactivity? In both NS and WS cell types?

Reviewer #6 brings up an interesting and important scientific question related to the data: does attenuating neuronal activity in hyperactive mice return activity levels to normal, wild type control levels? This is a question, among others, that we are actively investigating in experiments for a subsequent manuscript.

The chemogenetic experiments reported in our earlier Figures 4-5 were not designed to rigorously address the specific question of neuronal activity normalization in EC-Tau/hAPP mice. Instead, the experiments were designed to test the impact of attenuated EC neuronal activity on the accumulation of downstream, hippocampal Aβ and tau pathology. However, we are happy to report quantifiable changes in theta modulation and total spiking activity after CNO minipump implantation (Revised Figure 4A-B) and now show new data reporting reductions in the normalized average firing rates of Total EC neurons (Revised Figure 4C), Narrow-Spiking neurons (Revised Figure 4D) and Wide-Spiking neurons (Revised Figure 4E) at Week 6 of CNO-treatment versus Baseline. Please see our response to Reviewer #5 regarding this new data.

With that stated, we aimed to compare theta modulation and neuronal firing rates between groups as fairly as possible in order to approach the analysis requested by Reviewer #6. This resulted in three group comparisons of interest: WT Control, untreated (n=9 mice); EC-Tau/hAPP, untreated (n=10 mice); EC-Tau/hAPP, CNO-treated (Week 6) (n=8 mice). To examine the impact of CNO-treatment on theta modulation, we performed a One-Way ANOVA on % Theta Power values for mice in these conditions (Rebuttal Figure 3). We found that % Theta power was significantly reduced in CNO-treated EC-Tau/hAPP mice (Week 6) compared to untreated Controls ($p < 0.001$), but not compared to untreated EC-Tau/hAPP values ($p > 0.05$). % Theta power in untreated EC-Tau/hAPP mice was reduced compared to untreated Control mice ($p < 0.01$).

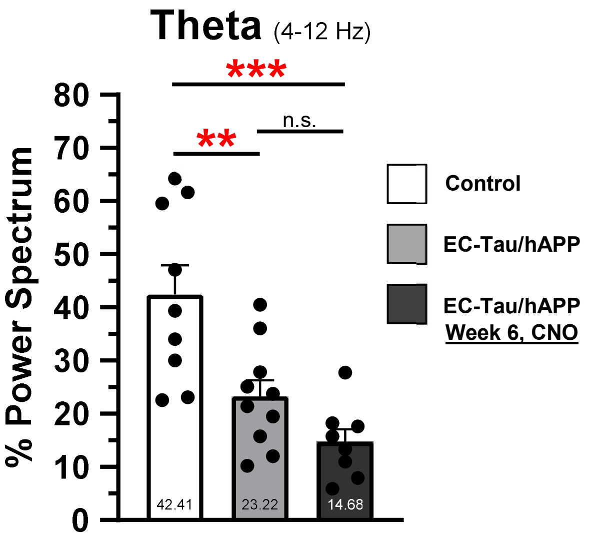

Rebuttal Figure 3. Chronic hM4Di EC DREADDs and % Theta Power. We compared the % Theta power values of 1mg/kg/day CNO-treated EC-Tau/hAPP mice (n=8) at Week 6 generated in Figure 4A to Control (n=9) and EC-Tau/hAPP (n=10) values reported in Figure 3C. We found that % Theta power was significantly reduced in CNO-treated EC-Tau/hAPP mice compared to untreated Control values, but not compared to untreated EC-Tau/hAPP % Theta power values. One-way ANOVA test: $F_{(2,24)} = 12.52$, $p < 0.001$. Tukey's multiple comparisons test: Week 6, CNO versus Control, $p < 0.001$; EC-Tau/hAPP versus Control, $p < 0.01$; Week 6, CNO versus EC-Tau/hAPP, n.s. Graphical representations appear as mean ± SEM. ** $p < 0.01$. *** $p < 0.001$.

After automatic spike sorting and manual cluster cutting of recording data at Week 6 of CNO-treatment, we calculated the total number of neurons collected (n=131 single-units) across eight mice and the average number of neurons collected per EC-Tau/hAPP mouse (n=16 single-units). To fairly compare the firing rate data from this Week 6 dataset to WT Control, untreated and EC-Tau/hAPP, untreated groups presented in Figure 2, we randomly selected n=16 single-units from each mouse after shuffling datasets 50X. This resulted in n=128 single-units in the WT Control, untreated group (n=8 mice) and n=128 single-units in the EC-Tau/hAPP, untreated group (n=8 mice), allowing a fair comparison based on the number of cells compared. As a reminder, the average firing rates reported in Figure 2 were generated from much larger datasets (Control: n=386 single-units, n=8 mice; EC-Tau/hAPP: n=588 single-units, n=8 mice) and cannot be fairly compared to EC-Tau/hAPP neuronal firing rates at Week 6 of CNO-treatment (n=131 single-units, n=8 mice).

Performing a one-way ANOVA followed by Tukey's multiple comparisons test, we found a significant reduction in the average firing rate of CNO-treated EC-Tau/hAPP mice compared to untreated EC-Tau/hAPP mice ($p < 0.001$), but not compared to WT Controls ($p > 0.05$) (Rebuttal Figure 4). As expected from our reported data in Figure 2B, we found a significant increase in firing rates of untreated EC-Tau/hAPP mice versus untreated Controls ($p < 0.05$).

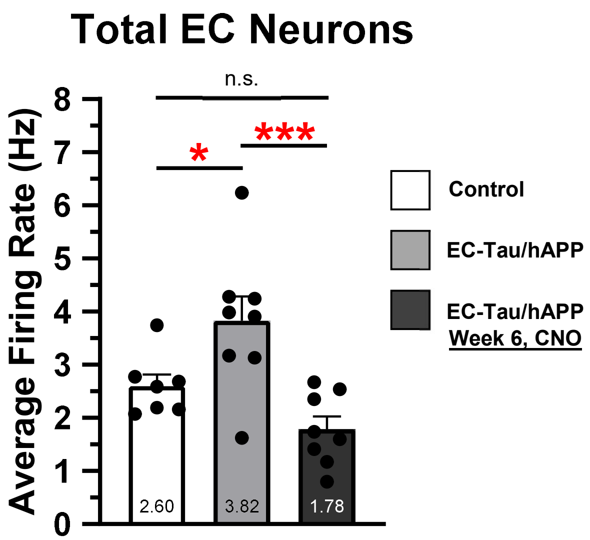

Rebuttal Figure 4. Chronic hM4Di EC DREADDs and neuronal firing rates. In order to fairly compare average firing rates of mice, we compared neuronal firing rates of 1mg/kg/day CNO-treated EC-Tau/hAPP mice (n=8) at Week 6 generated in Figure 4C to Control (n=7) and EC-Tau/hAPP (n=8) values reported in Figure 2B. We found that firing rates were significantly reduced in CNO-treated EC-Tau/hAPP mice compared to untreated EC-Tau/hAPP mice, but not compared to untreated Control mice. One-way ANOVA test: $F_{(2,20)} = 9.793$, $p < 0.01$. Tukey's multiple comparisons test: EC-Tau/hAPP versus Control, $p < 0.05$; Week 6, CNO versus EC-Tau/hAPP, $p < 0.001$. Graphical representations appear as mean ± SEM. * $p < 0.05$. *** $p < 0.001$.

These data optimistically suggest that the current CNO treatment regimen led to a "normalization" of theta activity and single-unit activity in EC-Tau/hAPP mice, as there were no significant post hoc differences in the appropriate group comparisons (e.g. AVG FRs, CNO-treated EC-Tau/hAPP mice versus untreated Control mice). However, we hesitate to draw these conclusions in the manuscript due to the limitations imposed on the experimental design. An experiment specifically designed to address this question would require higher density probes to record the maximum amount of EC neurons possible with no electrode movement, and the following treatment groups: WT Control, CaMKIIα-eGFP, 1mg/kg/day CNO; EC-Tau/hAPP, CaMKIIα-hM4Di-mCherry, 1mg/kg/day CNO; EC-Tau/hAPP, CaMKIIα-hM4Di-mCherry, Saline.

Visually, the data appears to be trending towards single-unit and network hypoactivity after chronic DREADDs-activation, though we cannot state with certainty that the current CNO treatment regimen led to EC hypoactivity in these mice, as neuronal firing rates were not significantly reduced compared to untreated Controls, and theta power was not significantly reduced from untreated EC-Tau/hAPP mice. The trends make it tempting to claim that chronic hM4Di EC DREADDs activation via CNO further reduced EC activity levels well-beyond normal, but better designed experiments specifically implanting controls testing for that need to be run.

Line 275. "CP27+ immunostaining appeared to be selectively decreased". Why is there no quantification? These sentences are unclear, what differences should be shown in Fig 5C, D?

Quantification was performed on CP27+ immunostained sections and is reported in Figure 5E. We did not detect statistically significant differences in right versus left hemisphere CP27+ immunostaining in our hippocampal ROIs: DG ($p = 0.1678$), CA1 ($p = 0.0740$) or Subiculum ($p = 0.1423$).

We agree that the sentence identified by Reviewer #6 (Line 275) is unclear given the quantified results, and so we have removed it from the revised manuscript.

Line 386 - " blunted hyperactivity in NS neurons of EC-Tau/hAPP mice (Figure 2E), which may represent an early, synergistic effect of tau on Aβ-associated inhibitory interneuron dysfunction that could precede subsequent impairments in excitatory neurons and gross network function". Indeed, the authors should suggest explanations for the blunted hyperactivity of putative interneurons in EC-Tau/hAPP mice. But I must say that the sentence above is quite unclear, and should at least be rephrased, and possibly split in several sentences.

We did not detect significant differences in Narrow-Spiking neuronal firing rates between 16-month EC-Tau/hAPP mice and age-matched Controls (Figure 2E). A potential mechanism that may explain this observation was discussed in a Response to Reviewer #5 (Critique #6), but is briefly discussed here as well. Jorge Palop's work demonstrating reduced Nav1.1 (voltage-gated sodium channel predominantly expressed in interneurons at the

action-initial segment) functionality offers a mechanistic clue as to the root of hyperactivity in our mouse model. Pathological tau in the EC-Tau/hAPP mice may impose additional challenges on ion channels at the axon-initial segment of interneurons by this time-point (16-months), reducing their spontaneous firing rates. Wide-Spiking EC neurons were still notably hyperactive in EC-Tau/hAPP mice relative to Controls (Figure 2F), though the mean firing rates calculated were somewhat variable. We know that advanced tau pathology affects excitatory grid cell function at 30+ months in the EC-Tau mouse line. Thus, we believe the difference in mean firing rates of Narrow Spiking EC neurons in EC-Tau/hAPP mice versus hAPP mice is reflective of the time-point sampled.

We are careful not to speculate too much about the mechanisms underlying single-unit or network dysfunction in our mice, as additional experiments are required to define exactly how Aβ and tau interact at the cellular level to impair neuronal activity. These effects are likely age-dependent, as well.

Finally, we concede that the sentence identified by the reviewer is quite unclear. We have now revised the statement, which appears as follows (Lines 601-04, clean manuscript version): "Our analysis in 16-month EC-Tau/hAPP mice revealed blunted hyperactivity in NS neurons (Figure 2E). This may represent a relatively early effect of $hTau^{P301L}$ on Aβ-associated interneuron dysfunction prior to subsequent impairment in excitatory neurons".

Reviewer #7 (new reviewer): Rodriguez and collaborators propose a convincing report that focuses on the functional relationship between co-expression of human APP/Tau and tau pathology in the EC-Hippocampal network. The chemogenetic approaches are undoubtedly fascinating methods to decrease neuronal activity and observe the resulting effects on abeta/tau pathology.

This Reviewer has appreciated the Author's efforts to ameliorating the manuscript and to adequately reply Reviewers requests. Thus, this Reviewer recommends the Ms for publication.

We thank Reviewer #7 for appreciating our efforts to improve our manuscript and recommending it for publication with *PLOS Biology* in its current form.
