# Supplementary material for: Chemogenetic attenuation of neuronal activity in the entorhinal cortex reduces Aβ and tau pathology in the hippocampus
Source: PLoS Biol. 2020 Aug 21;18(8):e3000851. doi: 10.1371/journal.pbio.3000851 (PMC7467290; doi:10.1371/journal.pbio.3000851)
Supplement: Attachment — Submitted filename: Review Plos Biology - Copy.docx [file pbio.3000851.s008.docx]

In this manuscript, Rodriguez et al. use a new transgenic mouse line expressing both Tau and hAPP in the entorhinal cortex (EC) to show that hAPP aggravates tau aggregation in the entorhinal cortex and accelerates pathological tau spread into the hippocampus. The authors identify hAPP/Aβ, and not tau, as the major trigger for neuronal hyperactivity and impaired theta in the EC. Finally, the authors use a chemogenetic approach to attenuate neuronal hyperactivity in the EC and they provide evidence for reduced hAPP/Aβ. accumulation and reduced pathological tau spread downstream in the hippocampus. Overall this study nicely complements previous studies showing abnormal activity patterns in the context of AD, and its link with disease progression.

It is a revision of a manuscript for which the main criticisms dealt with small sample size and with quantification methods. The authors have made the appropriate efforts to answer to most of the concerns of the reviewers. They have increased the sample size when necessary, and improved the presentation. The manuscript should now be potentially suitable for publication, provided minor revisions indicated below:

Figure 1A – It is quite unfortunate that there is no quantification of the plaques or hAPP/Aβ immunoreactivity, in the different areas and models as there is for MC1 immunoreactivity. See line 241, this has been done in part of the CNO experiments.

Figure 1D – What is the use of these panels, and what differences with middle panels in 1A? In addition, what is the meaning and utility of the thick black lines?

Figure 4 and 5. The effect of chemogenetic attenuation of EC neuronal activity on Aβ and Tau pathology is perhaps the most original result of the paper. The effect occurs on 16 months-old EC-Tau/hAPP mice which already exhibit significant level of pathology. A treatment with CNO for 6 weeks diminishes the level of both amyloid (Fig4D, E) and Tau (Fig5J, O). Thus, CNO does not inhibit the appearance of AD-related hallmarks but rather seems to partially revert the pathology. Surprisingly, there is no mention about the kinetic of the events and about the possible mechanisms involved: inhibition of Ab production and Tau phosphorylation, versus clearance of plaques and tangles. At minimum, this should be addressed in the discussion.

Line 226 – The authors should provide a comparison between the levels of neuronal activity (and theta power) attained after CNO treatment, and those observed in WT mice at the same age. Does the CNO treatment lead to normalization of neuronal activity? Or hypoactivity? In both NS and WS cell types?

Line 275. *“CP27+ immunostaining appeared to be selectively decreased”.* Why is there no quantification? These sentences are unclear, what differences should be shown in Fig 5C,D?

Line 386 – *“ blunted hyperactivity in NS neurons of EC-Tau/hAPP mice (Figure 2E), which may represent an early, synergistic effect of tau on Aβ-associated inhibitory interneuron dysfunction that could precede subsequent impairments in excitatory neurons and gross network function”*. Indeed, the authors should suggest explanations for the blunted hyperactivity of putative interneurons in EC-Tau/hAPP mice. But I must say that the sentence above is quite unclear, and should at least be rephrased, and possibly split in several sentences.
